# Gene dosage and protein valency impact phase separation and fungal cell fate

**DOI:** 10.1371/journal.pgen.1011810

**Published:** 2025-08-08

**Authors:** Collin Ganser, Peiling He, Corey Frazer, Damian J. Krysan, Richard J. Bennett

**Affiliations:** 1 Department of Molecular Microbiology and Immunology, Brown University, Providence, Rhode Island, United States of America; 2 Department of Pediatrics, Carver College of Medicine, University of Iowa, Iowa City, Iowa, United States of America; 3 Department of Molecular Physiology and Biophysics, Carver College of Medicine, University of Iowa, Iowa City, Iowa, United States of America; UT Health San Antonio: The University of Texas Health Science Center at San Antonio, UNITED STATES OF AMERICA

## Abstract

Cell fate decisions in eukaryotes are regulated by interconnected networks of transcription factors (TFs) that drive heritable changes in identity. However, much is unknown about how TFs act together to control cell fate, despite links to cellular dysfunction and disease when TF function is aberrant. Here, we addressed the interplay between TFs that control heritable switching in the diploid fungal pathogen *Candida albicans*. This species can propagate in two distinct cell states, white and opaque, with epigenetic transitions between states regulated by a core network of eight TFs plus >100 auxiliary TFs. The role of these TFs was dissected using simple and complex haploinsufficiency (CHI) analyses to examine the impact of gene dosage on cell fate. Among single heterozygotes, loss of one allele of *WOR1* had the greatest impact on white-opaque switching, consistent with its role as the master opaque regulator, while CHI analysis revealed strong genetic interactions between other core TFs including *WOR3* and *WOR4*. Wor1 function was also highly sensitive to its interaction valency, a measure of the number of inter-molecular interactions it can undergo. Engineered strains with increased Wor1 valency, either via the addition of extra prion-like domains (PrLDs) or by forced dimerization, increased switching frequencies by up to two orders of magnitude. Increasing Wor1 valency increased its propensity to form phase-separated condensates both *in vitro* and in mammalian cells. Together, these experiments establish that changes to TF gene dosage and TF valency can alter cell fate determination, with these changes linked to the propensity of TFs to undergo condensate formation.

## Author summary

There is a pressing need to understand the precise roles of TFs in regulating cell identity. The fungal pathogen *Candida albicans* undergoes transitions between two heritable states, white and opaque, regulated by an interconnected network of eight core TFs that control their own expression as well as that of each other. Here, we addressed the contributions of core and auxiliary TFs to white-opaque switching by performing simple and complex haploinsufficiency analyses in which heterozygous mutations were examined individually and in combination. We show that *WOR1* plays a unique role in white-opaque switching as *WOR1* heterozygotes exhibited an almost complete block in opaque cell formation. Moreover, alterations in Wor1 valency – which denotes the number of potential intermolecular interactions – led to corresponding changes in phase separation and function. We therefore define the individual and combinatorial contributions of *C. albicans* TFs to cell fate determination and demonstrate that changes to both TF gene dosage and valency can impact cell differentiation via the modulation of condensate properties.

## Introduction

Transcription factor (TF) networks play a central role in regulating cell fate across the tree of life, from bacteria to multicellular eukaryotes [1–3]. In microbial systems, transitions between alternative cell states can promote bet hedging to promote survival under fluctuating environmental conditions or can enable a division of labor between specialized cell types [4–6]. In higher organisms, cell fate transitions are associated with cell differentiation during development, cell reprogramming to generate pluripotent stem cells, and the formation of aberrant cell types during tumorigenesis [7,8]. Mammalian cell identity is determined by TFs that act together with cell signaling cues, chromatin structure, nucleosome remodeling and post-translational histone modifications to control cell differentiation [9–11].

Cell fate-defining TFs act at *cis*-regulatory elements to regulate target gene expression and, in higher eukaryotes, these elements can be more than 1 Mb away from their target [12]. Cell identify has also been linked to specialized super-enhancers where high levels of TFs, Mediator complex and RNA polymerase II reside together with elevated levels of active histone marks [7,12,13]. Assembly of the transcriptional machinery at super-enhancers may be enabled by constituent factors undergoing phase separation (liquid-liquid demixing or complex coacervation) into biomolecular condensates at these loci [7,12–14]. Models suggest that weak interactions between the intrinsically disordered domains of TFs, cofactors and RNA polymerase II, together with protein-nucleic acid interactions, drive the formation of transcriptional condensates that induce cell identity genes [15–19].

The transcriptional control of cell fate has been extensively investigated in *Candida albicans*, a fungal species that is of critical importance to human health [20–23]. This species is a human commensal but can cause localized or systemic infections upon immunosuppression, microbiome disruption or host epithelial cell damage [20–22]. *C. albicans* exhibits phenotypic plasticity which can enable adaptation to host niches and can promote immune evasion [24–28]. In particular, this species undergoes stochastic and reversible switching between two stable cell states, white and opaque [29–31], with switching sensitive to a range of environmental cues including N-acetylglucosamine and CO_2_ [32–35].

Regulation of the white-opaque switch shows several parallels with the control of cell differentiation in higher eukaryotes. White-opaque switching is controlled by over 100 TFs with a core transcriptional regulatory network (TRN) of eight TFs that bind to their own and to each other’s promoters and that operate in a series of nested feedback loops [36–39]. *WOR1* is regarded as the master regulator of the switch; cells lacking *WOR1* generally cannot switch to the opaque state while *WOR1* overexpression drives cells to the opaque state *en masse* [40–42]. Similar to cell fate regulation in mammalian cells, the core white-opaque TFs bind to unusually large regulatory regions that may recruit high levels of the transcriptional machinery [36,37,39,42]. Moreover, most core white-opaque TFs possess intrinsically disordered prion-like domains (PrLDs) that can promote condensate formation via their multivalency, and mutations that block condensate formation block TF function in *C. albicans* cells [43]. It is therefore envisaged that, as in mammalian super-enhancers, core white-opaque TFs assemble into condensates at key genomic regions to drive gene expression and cell identity [43]. As in higher eukaryotes, white-opaque TFs also cooperate with chromatin-modifying activities to direct cell fate [36–39,42,44–49].

Here, simple and complex haploinsufficiency (CHI) analyses were performed on *C. albicans* TFs to examine genetic interactions that impact white-opaque switching. CHI analyses involves the construction of strains with heterozygous deletions at two separate loci [50,51], and enables genetic interactions to be evaluated even when one TF is essential to a process [50]. Notably, we show that *WOR1* heterozygotes exhibit an almost complete block in white-to-opaque switching, establishing the unique role of Wor1 in this transition, while CHI analyses identified complex epistatic interactions between other core TFs. Moreover, we show that changes to TF valency – reflecting the interaction potential of a given protein – can radically alter Wor1 function and its propensity to undergo liquid-liquid demixing. Thus, forcing Wor1-Wor1 dimerization altered the ability of this TF to undergo phase separation and increased white-to-opaque switching frequencies by almost two orders of magnitude. Together, these data highlight that changes to gene dosage and interaction valency can markedly alter TF function due to changes in phase separation.

## Results

### Analysis of heterozygous TF deletions for white-opaque switching

Genetic interactions between the core white-opaque TFs were examined using both simple and complex haploinsufficiency (CHI) analyses, in which *C. albicans* strains were heterozygous for one or two genes, respectively. Switching frequencies were evaluated in *MTL***a**/**a** or *MTL***a**/Δ derivatives of SC5314 *MTL***a**/α cells, as **a**/α cells are restricted from undergoing switching due to inhibition via *MTL***a**1/α2 [52–54]. Unless otherwise stated, switching experiments were performed by culturing white cells on synthetic complete dextrose (SCD) supplemented with 5 µg/mL Phloxine B and grown at 25°C for 7 days, conditions that support ~3% stochastic switching to the opaque state. Where appropriate, we also utilized conditions that promoted higher white-to-opaque switching frequencies including incorporation of N-acetyl glucosamine (GlcNAc) into the media and/or incubation in the presence of 5–20% CO_2_ [32,33].

**Fig 1:**
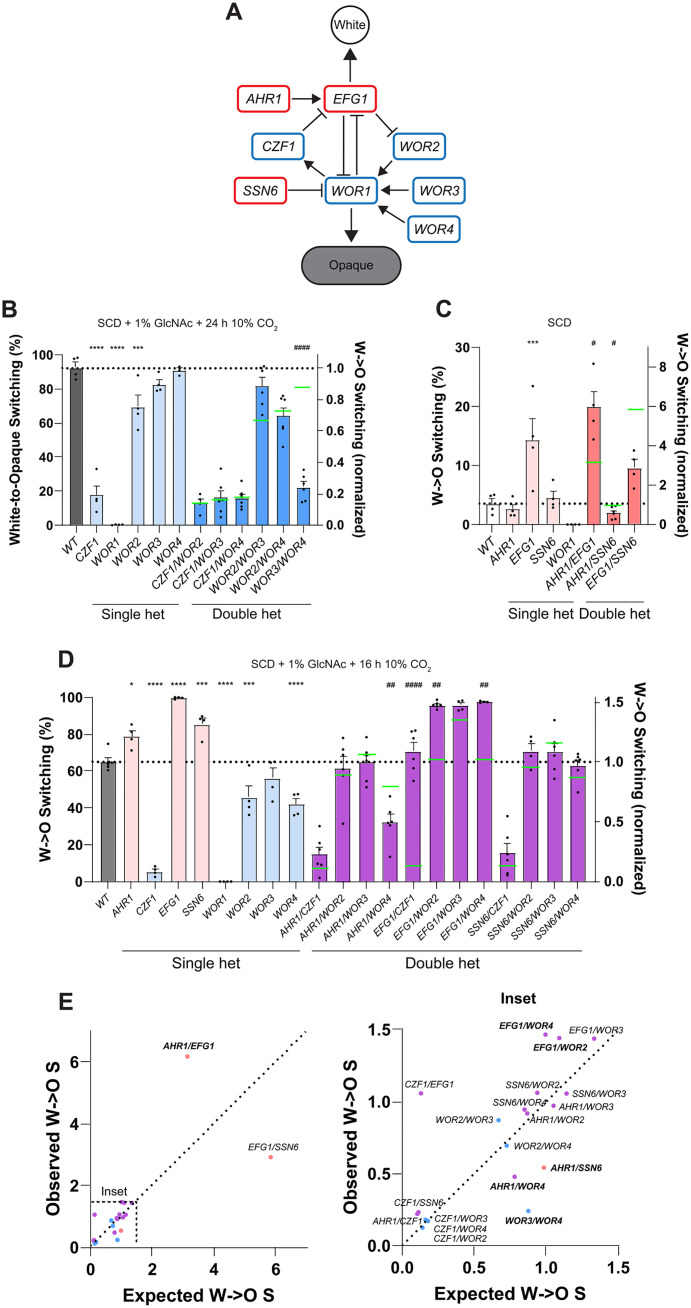
White-to-opaque switching frequencies of core white-opaque TF heterozygotes. **(A)** Core TFs regulating white-opaque switching. TFs in blue promote the opaque state and TFs in red promote the white state. Adapted from [28]. **(B-D)** Strains were grown on **(B)** SCD with 1% GlcNAc + 24 h 10% CO_2_ before outgrowth in normoxia, **(C)** SCD, or **(D)** SCD with 1% GlcNAc + 16 h 10% CO_2_ before outgrowth in normoxia. All experiments were performed for 7 days at 25°C. Mean white-to-opaque switching percentages are shown on the left y-axis and mean normalized white-to-opaque switching frequencies are shown on the right y-axis relative to the wildtype (WT) control. Gray/white bars represent WT strains. Light blue bars and red bars represent single heterozygotes for positive and negative regulators, respectively. Dark blue bars and dark red bars represent double heterozygotes for positive and negative regulators, respectively. Dark purple bars represent double heterozygotes with one negative and one positive regulator. Green lines indicate expected switching frequencies of double heterozygotes based on multiplying the switching frequencies of the corresponding single heterozygotes. Black dots indicate biological replicates, error bars show standard error of the mean (SEM), and the dotted line corresponds to WT. Statistical analysis was performed using ordinary one-way ANOVA with Dunnett’s multiple-comparison test, in which normalized switching frequencies of single heterozygotes were compared to that of the WT control. *P < 0.05; **P < 0.01; ***P < 0.001; ****P < 0.0001. Statistical analysis was also performed using a two-tailed Student’s T-test, in which the normalized switching frequencies were compared between the observed double heterozygote and that expected by multiplying single heterozygote values. #P < 0.05; ##P < 0.01; ####P < 0.0001. **(E)** Normalized switching frequencies of observed vs. expected double heterozygote strains. Double heterozygote strains that exhibited switching frequencies that were significantly different from their expected switching frequencies (based on multiplying the switching frequencies of the corresponding single heterozygotes) are shown in bold.

The core white-opaque transcriptional regulatory network (TRN) consists of five positive regulators of the opaque state (*CZF1*, *WOR1*, *WOR2*, *WOR3*, and *WOR4*), and three negative regulators of the opaque state (*AHR1*, *EFG1*, and *SSN6*) (Fig 1A). Analysis of the eight core TF heterozygotes showed that loss of one allele of *CZF1*, *WOR1* or *WOR2* significantly reduced white-to-opaque switching (Fig 1B and 1D). For example, under conditions where the control strain exhibited switching in >90% of colonies, switching was observed in 17.6% of *CZF1* heterozygote colonies, 69.5% of *WOR2* heterozygote colonies and in just 0.3% of *WOR1* heterozygote colonies (Fig 1B). These results are consistent with studies showing that the complete loss of each of these genes blocks or substantially reduces white-to-opaque switching [39,41,42,55]. Conversely, deletion of one allele of *EFG1* or *AHR1* significantly increased switching (Fig 1C and 1D), consistent with reports that reducing *EFG1* gene dosage enhances switching to opaque [55,56] and that a complete loss of *AHR1* (also called *ZCF37*) increases switching [57,58]. Loss of one allele of *WOR4* significantly decreased switching to opaque while the loss of one allele of *SSN6* significantly increased switching under a subset of conditions (Fig 1D). These results were unexpected since heterozygous deletion mutants of these genes have previously been reported to switch at normal frequencies [37,38]. Finally, loss of one allele of *WOR3* did not impact switching frequencies (Fig 1B and 1D), consistent with *wor3*Δ/Δ mutants showing normal white-to-opaque switching [44].

**Fig 2:**
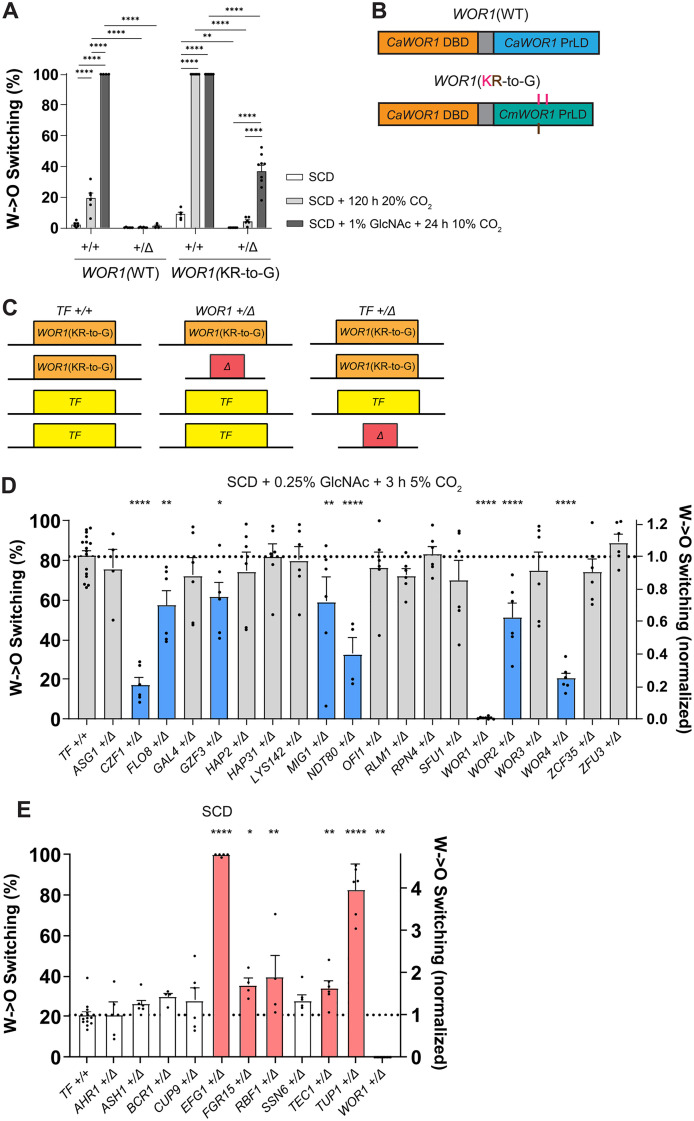
White-to-opaque switching frequencies of single TF heterozygotes in a *WOR1*(KR-to-G) strain background. **(A)** Switching assays were performed with WT *WOR1* or a KR-to-G variant of *WOR1*. *WOR1 + */+ and *WOR1* + /Δ heterozygote strains were plated on SCD (white bars), SCD with growth in 20% CO_2_ for 120 h before outgrowth in normoxia (light gray bars), or SCD with 1% GlcNAc and growth in 10% CO_2_ for 24 h before outgrowth in normoxia (dark gray bars). Switching percentages were determined after growth at 25°C for 7 days. Mean white-to-opaque switching percentages are shown; black dots indicate biological replicates and error bars show SEM. Statistical analysis was performed using ordinary one-way ANOVA with Dunnett’s multiple-comparison test, in which switching percentages were compared within *WOR1*(WT) or *WOR1*(KR-to-G) backgrounds, and between *WOR1 + */+ and *WOR1* + /Δ strains. **P < 0.01; ****P < 0.0001. **(B)** Schematics of WT Wor1 consisting of *C. albicans* DNA-binding domain (DBD) and PrLD and the *WOR1*(KR-to-G) variant which consists of the *C. albicans* DBD and a *C. maltosa* PrLD containing three KR-to-G substitutions (as indicated). **(C)** Genotypes of the strains used in **2D + E**. *TF + / +* strains contained two *WOR1*(KR-to-G) alleles, *WOR1* + /Δ strains contained one copy of *WOR1*(KR-to-G) and two copies of all other TFs, and *TF + /*Δ strains contained two copies of *WOR1*(KR-to-G) but one copy of the corresponding TF. **(D + E)** *TF + /+* (WT) and *TF + /*Δ (single heterozygotes) in the *WOR1*(KR-to-G) background were grown on **(D)** SCD with 0.25% GlcNAc in 5% CO_2_ for 3 h before outgrowth in normoxia or **(E)** SCD. Switching percentages were determined after growth at 25°C for 7 days. Mean white-to-opaque switching percentages are shown on the left y-axis and mean normalized white-to-opaque switching frequencies are shown on the right y-axis relative to WT from same-day experiments. Black dots indicate biological replicates, error bars show SEM, and the dotted line corresponds to the control strain’s switching frequency. Colored bars indicate significant differences from the WT control; blue for decreased switching and red for increased switching. Statistical analysis was performed using ordinary one-way ANOVA with Dunnett’s multiple-comparison test, in which switching was compared between mutants and the control. *P < 0.05; **P < 0.01; ****P < 0.0001.

We focused on the very low switching observed in *WOR1* heterozygotes, as even under strong opaque-inducing conditions (100% switching in control cells) these cells exhibited only ~1.1% switching, indicating a two orders of magnitude reduction (Fig 2A). Strains lacking either the A or B allele of *WOR1* were evaluated, as the two alleles differ by two residues in SC5314 (G125A and S633N; S1A Fig). When grown on strong opaque-inducing conditions (SCD + 1% GlcNAc + 10% CO_2_ for 24 h before outgrowth in normoxia), *WOR1* heterozygotes containing the A allele exhibited 5.2% switching whereas those harboring the B allele showed 0.5% switching, indicative of a 10-fold difference in activity (S1B Fig). Together, these experiments establish that *WOR1* gene dosage has an outsized role in determining white-opaque switching frequencies regardless of which *WOR1* allele is deleted, although differences in activity exist between the two SC5314 *WOR1* alleles.

To determine the impact of TF gene dosage on RNA expression, quantitative real time-PCR (qRT-PCR) was performed on TF heterozygotes in the white and opaque states. Most heterozygote strains showed TF expression levels that were 2-fold (or more) lower than the parental strain in both cell states (S2A Fig), although white *WOR1* + /Δ cells had slightly elevated (~1.5-fold higher) *WOR1* expression levels than the WT control (S2A Fig). These results are in line with gene expression generally correlating with gene dosage [51,59]. We also note that *EFG1* expression was higher in white cells than opaque cells, whereas *CZF1*, *WOR1*, *WOR2*, and *WOR3* were expressed at higher levels in opaque cells than in white cells (S2B Fig). *AHR1*, *SSN6*, and *WOR4* were expressed at similar levels between white and opaque cells (S2B Fig). These data align with RNA-seq analysis of gene expression levels in white and opaque cells [60].

### Complex haploinsufficiency (CHI) reveals genetic interactions between TFs

To identify genetic interactions between core white-opaque TFs, CHI analysis was performed by constructing double heterozygous TF mutants and comparing switching frequencies with the corresponding single TF heterozygotes. As noted above, switching frequencies with *WOR1* + /Δ heterozygotes were extremely low which precluded CHI analysis, a point returned to below. CHI analysis for the seven other core network genes involved construction of 21 different *TFA* + /Δ *TFB* + /Δ combinations. Three different culture conditions were used: SCD + 1% GlcNAc + 24 h 10% CO_2_ (which induces high levels of switching; Fig 1B), SCD (which supports low levels of stochastic white-to-opaque switching; Fig 1C), and SCD + 1% GlcNAc + 16 h 10% CO_2_ (for moderate levels of switching; Fig 1D).

For this analysis, we used the multiplicative model of genetic interactions to examine the data [50,51,61]. Briefly, the quantitative phenotype of the double mutant (*TFA* + /∆ *TFB* + /∆), in this case the ratio of the switching frequency normalized to a WT control (SF_AB_), was compared to the product of the phenotypes from the corresponding single mutants (SF_A_ x SF_B_). If SF_AB_ > SF_A_ x SF_B_, then a positive genetic interaction exists whereas if SF_AB_ < SF_A_ x SF_B_, then a negative interaction exists. If SF_AB_ = SF_A_ x SF_B_, then there is no genetic interaction and the two genes function independently. Positive interactions are frequently referred to as suppressive interactions and imply that the two genes function in a linear pathway or that a compensatory response is involved in the phenotype of the double mutant. Negative interactions suggest that the two genes function cooperatively, such as in a “synthetic lethal” phenotype. Even the observation of “no interaction” is informative as it indicates that two genes affect a phenotype independently of one another. We also note that very high or very low switching frequencies could limit the accuracy of certain CHI comparisons, such as that of *EFG1* heterozygotes which showed switching in 99.6% of colonies in some assay conditions (Fig 1D).

Here, most of the 21 double heterozygote mutants showed white-to-opaque switching frequencies equivalent to the product of the individual heterozygote switching frequencies, indicating that most TFs work independently to regulate the switch (Fig 1) [51,61]. The rate of switching for each double mutant was plotted against the product of the two corresponding single heterozygote mutants (SF_A_ x SF_B_, Fig 1E). Double mutants that lie close to the diagonal are those with independent contributions from each TF. Despite the large number of potential interactions based on the binding-data derived network [36–38], only six TF x TF interactions were significant by CHI (Fig 1E), indicating that most TFs make independent contributions to the white-opaque switch. A negative interaction between *WOR3* and *WOR4* indicates that these two factors function cooperatively to promote switching to opaque. Loss of one allele of *EFG1* in combination with loss of one allele of *WOR2* or *WOR4* resulted in close to 100% switching, similar to that of the single *EFG1* heterozygote, indicating that the change in *EFG1* dosage had a larger effect on switching than the change *WOR2* or *WOR4* dosage (Fig 1D). A positive interaction between *AHR1* and *EFG1* was observed indicating that they work cooperatively to inhibit switching to the opaque state.

We further investigated the interactions between *WOR3* and *WOR4* by analyzing single and double heterozygotes for *WOR1*, *WOR3* and *WOR4* expression. Although small (~2-fold) differences in expression of these genes were observed, none of these differences were significant (S2C Fig). This suggests that *WOR3* and *WOR4*, either individually or in combination, do not influence white-opaque switching by changes to *WOR1* expression.

### Identification of a hyperactive *WOR1* variant

**C. albicans* WOR1* heterozygote cells exhibited remarkably low white-to-opaque switching frequencies which impeded CHI analyses. To circumvent this issue, a hyperactive Wor1 variant was identified by mutation of the PrLD. Strains expressing the *C. albicans* Wor1 DNA binding domain fused to the *C. maltosa* Wor1 PrLD showed comparable activity to native *C. albicans* Wor1 in white-opaque switching assays (S3 Fig). Notably, however, substitution of the three positively charged residues in the *C. maltosa* PrLD generated a hyperactive *WOR1*(KR-to-G) variant (Fig 2A, 2B, and S3 Fig), consistent with previous studies showing that PrLD alterations can alter Wor1 function [43]. Thus, under conditions where one copy of the native *WOR1* allele supported only 1.1% white-to-opaque switching, a strain with one *WOR1*(KR-to-G) allele supported 37% switching to the opaque state (Fig 2A). As with the native *WOR1*, strains expressing one *WOR1*(KR-to-G) allele still showed much lower switching frequencies than strains expressing two alleles (4.3% vs. 100% switching in SCD + 20% CO_2_; Fig 2A). This again indicates that the white-opaque switch is extremely sensitivity to *WOR1* gene dosage.

### CHI reveals genetic interactions between *WOR1* and other white-opaque TFs

To investigate genetic interactions between *WOR1* and other white-opaque TFs (seven core TFs plus 21 auxiliary TFs [58]), we first generated single TF heterozygotes in a strain with two hyperactive *WOR1*(KR-to-G) alleles and evaluated white-to-opaque switching frequencies (Fig 2C). When grown under opaque-inducing conditions (SCD + 0.25% GlcNAc and 5% CO_2_ for 3 h before outgrowth in normoxia), the parental strain showed 82.6% switching while removal of one allele of the core TFs *CZF1*, *WOR2* or *WOR4,* or of the auxiliary TFs *FLO8*, *GZF3*, *MIG1* or *NDT80,* resulted in significantly decreased switching (Fig 2D). *CZF1* and *WOR4* heterozygotes showed the biggest defects of the core TFs with 17.6% and 20.7% switching, respectively, whereas the *NDT80* heterozygote had the largest defect of the auxiliary TFs with 32.9% switching (Fig 2D). Conversely, when grown on SCD without inducing cues, loss of one copy of *FGR15*, *RBF1*, or *TEC1* resulted in significant increases in switching, with 20.7% switching in the parental strain increasing to 34–39% switching in the heterozygotes (Fig 2E). The result with *RBF1* was surprising as a full *RBF1* deletion previously showed significantly decreased switching to the opaque state [58]. However, these differences were modest compared to those seen in *EFG1* and *TUP1* heterozygotes which showed 99.7% and 82.4% switching, respectively (Fig 2E). For comparison, we examined several auxiliary TFs in a WT *WOR1* strain background and found that heterozygotes affected switching as in the *WOR1*(KR-to-G) background. Thus, loss of one allele of *NDT80* reduced switching, loss of one allele of *TUP1* increased switching, and loss of one allele of *ASH1*, *RLM1*, or *RPN4* did not affect switching (S4 Fig).

**Fig 3:**
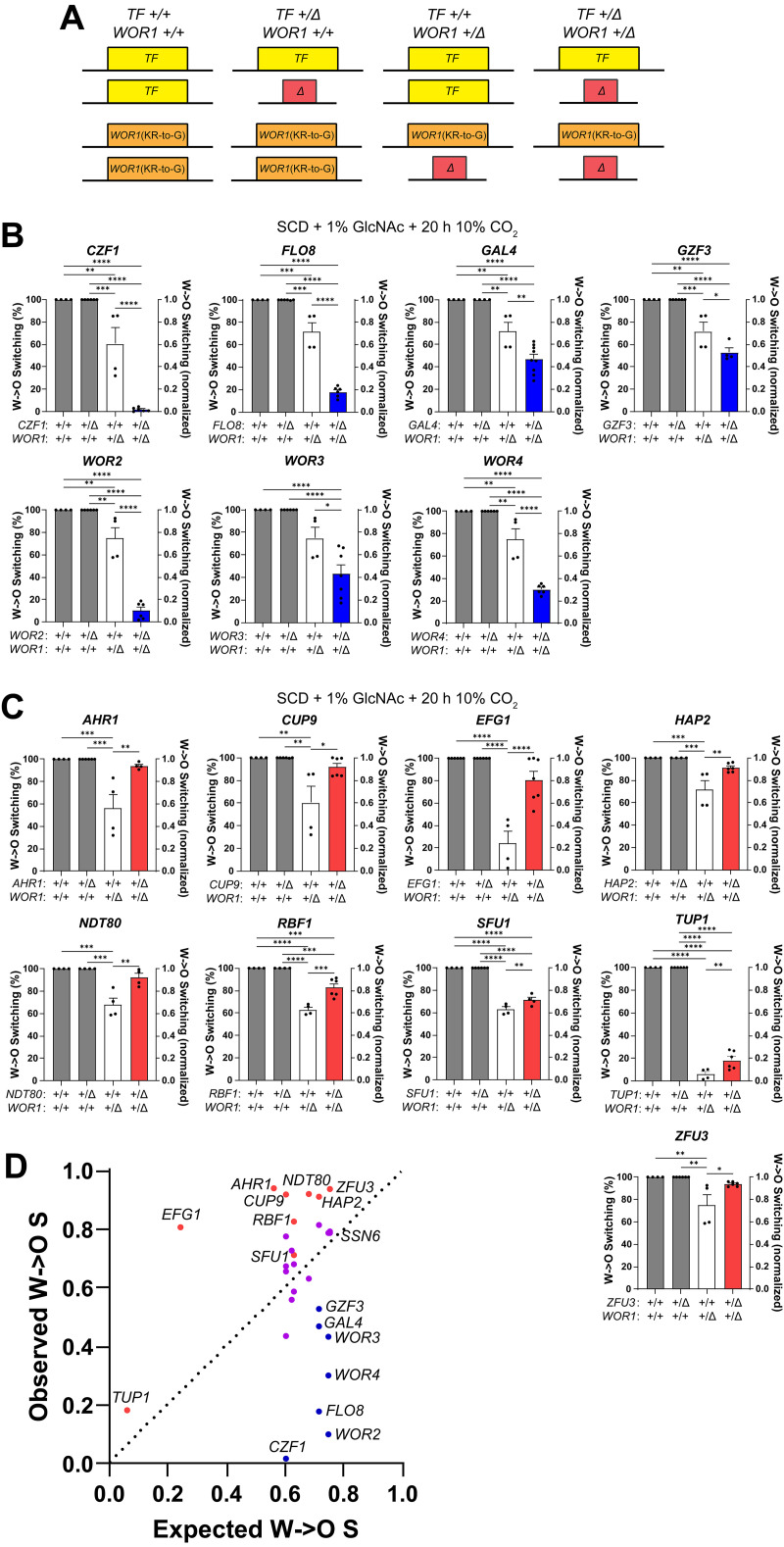
White-to-opaque switching frequencies of double TF heterozygotes in a *WOR1*(KR-to-G) strain background. **(A)** Genotypes of the strains used in **B + C**. **(B + C)** WT, single heterozygotes and double heterozygotes in the *WOR1*(KR-to-G) strain background were incubated on SCD + 1% GlcNAc in 10% CO_2_ for 20 h before outgrowth under normoxia. Switching frequencies were determined after growth at 25°C for 7 days. Mean white-to-opaque switching percentages are shown on the left y-axis and mean normalized white-to-opaque switching frequencies are shown on the right y-axis relative to the control from same-day experiments. Black dots indicate biological replicates and error bars show SEM. WT and TF single heterozygotes are shown in gray, *WOR1* single heterozygotes are shown in white, double heterozygotes that increased switching relative to the *WOR1* single heterozygote are shown in red and double heterozygotes that decreased switching relative to the *WOR1* single heterozygote are shown in blue. Statistical analysis was performed using ordinary one-way ANOVA with Dunnett’s multiple-comparison test, in which switching frequencies were compared between each strain. *P < 0.05; **P < 0.01; ***P < 0.001; ****P < 0.0001. **(D)** Normalized switching frequencies of observed vs. expected double heterozygote strains. Double heterozygote strains that exhibited switching frequencies that were significantly higher than their expected switching frequencies (positive interactions) are shown in red and those significantly lower (negative interactions) are shown in blue (based on multiplying the switching frequencies of the corresponding single heterozygotes).

We next analyzed double TF heterozygote mutants in the *WOR1*(KR-to-G) strain background (Fig 3A and S5A Fig). Under strong opaque-inducing conditions (SCD + 1% GlcNAc and 10% CO_2_ for 20 h before outgrowth in normoxia), all TF heterozygotes switched from white to opaque at 100% efficiency when two *WOR1*(KR-to-G) alleles were present, whereas analysis in the *WOR1*(KR-to-G) heterozygote showed 6–72% switching (Fig 3B, 3C, and S5B Fig). CHI analysis showed that *WOR1* worked cooperatively with the core positive regulators *CZF1*, *WOR2*, *WOR3*, and *WOR4,* as double heterozygotes with these genes switched significantly less than the single heterozygotes (Fig 3B and 3D). The *SSN6*/*WOR1* double heterozygote switched at comparable frequencies to the *WOR1* single heterozygote, indicating that *WOR1* was epistatic to *SSN6* (S5B Fig). Deletion of one allele of the negative regulators *EFG1* or *AHR1* in combination with deletion of one allele of *WOR1* resulted in switching frequencies between those of the single heterozygotes (Fig 3C and 3D), supporting the model that *EFG1* and *AHR1* work in opposition to *WOR1* in white-opaque switching.

Given central roles for *WOR1* and *EFG1* in switching, we further examined *WOR1-EFG1* interactions under additional conditions that supported 27–90% switching in the WT control. In these assays, the *WOR1/EFG1* double heterozygote generally switched at a frequency close to that of the *WOR1* heterozygote (S6A and S6B Fig). For example, in assays performed on SCD with 1% GlcNAc and 10% CO_2_, the WT strain switched at 90%, the *EFG1* heterozygote switched at 100% and the *WOR1* heterozygote switched at 0.1%, respectively (S6B Fig). Under these conditions, the *EFG1/WOR1* double heterozygote showed only 0.5% switching, close to that of the *WOR1* single heterozygote. This indicates that the loss of one *WOR1* allele had a dominant effect on switching over the loss of one *EFG1* allele. We also examined the impact of *WOR1* dosage in cells both with and without *EFG1* and found that *WOR1* copy number affected switching even in the absence of *EFG1* (S6C Fig).

Examination of *WOR1* interactions with auxiliary TFs revealed that *WOR1* works cooperatively with *FLO8*, *GAL4*, and *GZF3*, as their corresponding double heterozygotes switched less than the *WOR1* heterozygote (Fig 3B and 3D). In contrast, *CUP9*, *HAP2*, *NDT80*, *RBF1*, *SFU1*, *TUP1*, and *ZFU3* were antagonistic to *WOR1*, with the corresponding double heterozygotes switching more than the *WOR1* heterozygote (Fig 3C and 3D). The result with *NDT80* was surprising as this is a positive regulator of the opaque state (Fig 2D and [58]), and yet the *NDT80*/*WOR1* double heterozygote showed increased switching relative to the *WOR1* single heterozygote. Double heterozygotes of *WOR1* with the auxiliary TFs *ASG1*, *ASH1*, *BCR1*, *FGR15*, *HAP31*, *LYS142*, *MIG1*, *OFI1*, *RLM1*, *RPN4*, *TEC1*, and *ZCF35* showed switching frequencies comparable to the *WOR1* single heterozygote (Figs 3D and S5B). These results reveal positive, negative and neutral interactions between *WOR1* and auxiliary TFs (Fig 3D) and that *WOR1* was again the most influential TF in determining white-to-opaque switching frequencies under each condition.

### Increasing Wor1 valency with extra PrLDs increases white-to-opaque switching

**Fig 4:**
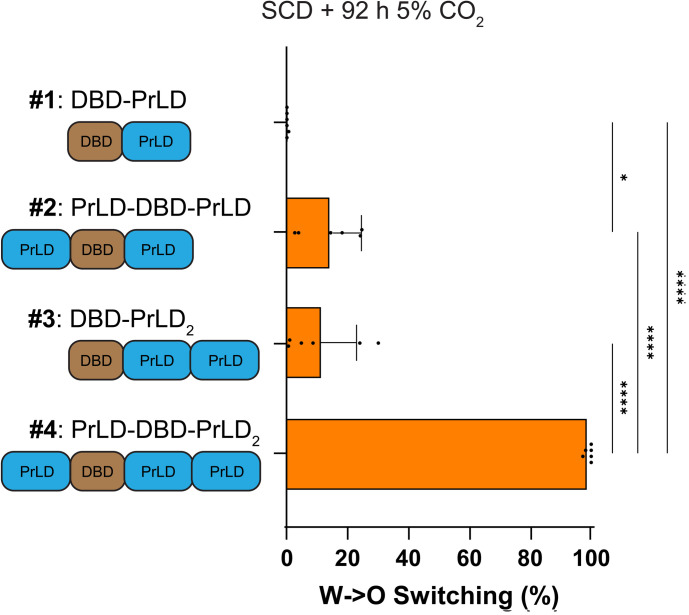
White-to-opaque switching frequencies of Wor1-PrLD variants. Schematics of Wor1 variants containing different PrLD configurations on the left. Construct #1 is the native Wor1 protein, constructs #2 and #3 have one extra Wor1 PrLD attached to the N- or C-terminus, respectively, and construct #4 has one extra Wor1 PrLD attached to both termini. Strains expressing each of the four Wor1 variants were incubated on SCD medium in the presence of 5% CO_2_ for 92 h before outgrowth in normoxia. Switching frequencies were determined after growth at 25°C for 7 days. Mean white-to-opaque switching percentages are shown; black dots indicate biological replicates and error bars show SEM. Statistical significance was evaluated using ordinary one-way ANOVA with Dunnett’s multiple-comparison test, in which all mean switching percentages were compared between each other. *P < 0.05; ****P < 0.0001.

Our results highlight that Wor1 plays a unique role in white-opaque switching. This TF can undergo phase separation due to weak multivalent interactions with itself via its PrLD or due to interactions with the PrLDs of other TFs [43]. Here, we examined the effect of modulating valency by adding extra copies of the Wor1 PrLD to the native protein. Four constructs were expressed in a *C. albicans wor1*Δ/Δ strain: 1) WT Wor1 (containing the DNA binding domain and a single PrLD; DBD-PrLD), 2) Wor1 with an extra N-terminal PrLD (PrLD-DBD-PrLD), 3) Wor1 with an extra C-terminal PrLD (DBD-PrLD_2_), and 4) Wor1 with extra N- and C-terminal PrLDs (PrLD-DBD-PrLD_2_) (Fig 4). When grown on SCD, the strain expressing WT Wor1 supported minimal (~0.1%) switching whereas strains expressing Wor1 with one extra copy of the PrLD (PrLD-DBD-PrLD and DBD-PrLD_2_) showed 11–14% switching (Fig 4). Remarkably, the strain expressing the Wor1 construct with two extra PrLDs (PrLD-DBD-PrLD_2_) showed almost 100% switching under the same culture conditions (Fig 4).

These Wor1 variants were expressed fused to mNeonGreen and all constructs were expressed within 20% of the wildtype Wor1-mNeon level (S7A–S7C Fig). White-to-opaque switching frequencies of the mNeonGreen-tagged Wor1 variants showed that the presence of extra PrLDs again resulted in higher white-to-opaque switching frequencies (S7D Fig), as in the untagged strains. Together, these experiments establish that the addition of extra PrLDs (and increased protein valency) greatly increases Wor1’s ability to promote white-to-opaque switching.

### Increasing Wor1 valency via dimerization increases white-to-opaque switching

**Fig 5:**
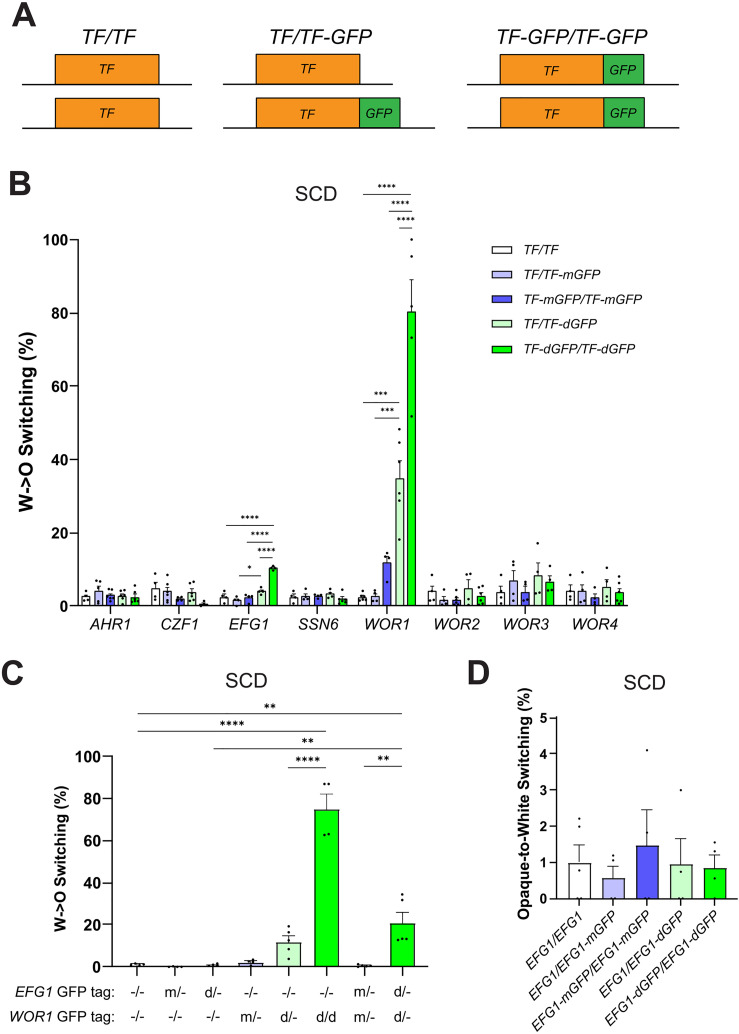
White-opaque switching frequencies of strains harboring GFP-tagged TFs. **(A)** Schematic showing TFs with alleles tagged with GFP. **(B)** Strains with TF alleles tagged by mGFP or dGFP were grown on SCD medium for 7 days at 22°C and white-to-opaque switching percentages determined. **(C)** Strains with TF alleles untagged (Ø) or tagged with mGFP (m) or dGFP (d) were grown on SCD medium for 7 days at 22°C and white-to-opaque switching percentages determined. **(D)** Efg1-GFP strains were grown on SCD medium for 7 days at 25°C and opaque-to-white switching percentages determined. Mean switching percentages are shown; black dots indicate biological replicates and error bars show SEM. Blue bars indicate monomeric GFP tags and green bars represent dimeric GFP tags. Statistical analysis was performed using ordinary one-way ANOVA with Dunnett’s multiple-comparison test, in which mean switching percentages among strains for one TF were compared between each other. *P < 0.05; ***P < 0.001; ****P < 0.0001.

Previous studies showed that Wor1 fusion to dimerizing GFP (dGFP) resulted in white-to-opaque switching at a frequency 27-fold higher than fusion to monomeric GFP (mGFP) [62]. mGFP and dGFP differ by only a single point mutation, A206K, and the increased oligomerization of Wor1-dGFP over Wor1-mGFP was proposed to underlie its hyperactive function [62]. Given that Wor1 has also been shown to undergo phase separation [43], we hypothesized that Wor1 dimerization would increase Wor1 valency leading to enhanced phase separation and higher TF activity. To test this, we evaluated strains expressing Wor1-dGFP and Wor1-mGFP (Fig 5A) and, similar to Ziv *et al. [*62*],* Wor1-dGFP-expressing strains exhibited significantly higher switching frequencies than either untagged or Wor1-mGFP-expressing strains. For example, on SCD medium, a *WOR1*/*WOR1-*mGFP strain showed 2.6% switching whereas a *WOR1/WOR1-*dGFP strain showed 35% switching, a 13.5-fold difference (Fig 5B). Tagging both copies of *WOR1* with mGFP or dGFP also resulted in higher switching in the dGFP-tagged strain (80.4%) than in the mGFP-tagged strain (11.9%) (Fig 5B). These experiments establish that fusion of Wor1 to dGFP causes a substantial increase in switching whereas fusion to mGFP results in an insignificant change compared to the untagged control.

We evaluated whether tagging of other network TFs with mGFP/dGFP also impacts white-opaque switching. GFP tagging of most TFs did not alter switching, although Efg1-dGFP strains showed slightly increased white-to-opaque switching frequencies compared to untagged or Efg1-mGFP-expressing strains (10.3% vs. 2.2-2.5%, respectively; Fig 5B). We also tested strains with one allele of both *EFG1* and *WOR1* GFP-tagged to investigate potential synergy between these TFs. The strain co-expressing Efg1-dGFP Wor1-dGFP exhibited elevated switching relative to controls, but these levels were similar to a strain expressing only Wor1-dGFP (Fig 5C). Since Efg1 promotes the white state, we also tested the opaque-to-white switching frequency of an Efg1-dGFP strain but found no differences with control strains (Fig 5D).

TF expression levels were compared between strains in which both TF alleles were mGFP- or dGFP-tagged and, in general, TF expression levels were in line with expectations, with Wor1 being the highest expressed TF in opaque cells and Efg1 being the highest expressed TF in white cells (S8 Fig). We also found that most TFs showed similar expression levels between mGFP- and dGFP-tagged strains, although dGFP-tagging of Efg1 resulted in lower expression levels in white cells than tagging with mGFP (S8 Fig). This may contribute to the increased white-to-opaque switching observed in the Efg1-dGFP-tagged strain given that reduced *EFG1* expression increases switching to the opaque state (Fig 1C and 1D).

### Increasing TF valency leads to increased phase separation

**Fig 6:**
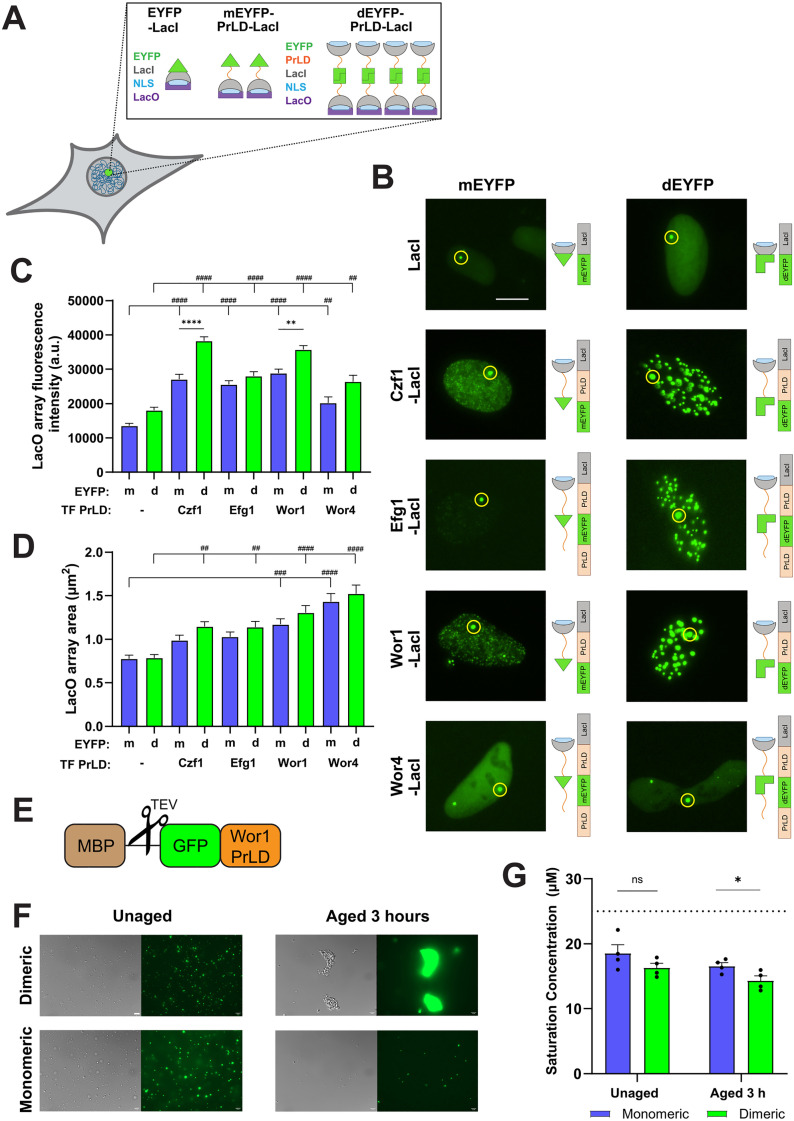
Dimerization of TFs promotes the formation of phase-separated condensates. **(A)** Schematic of EYFP-LacI variants, with or without TF PrLDs, that are recruited to LacO arrays to form fluorescent foci in U2OS nuclei. mEYFP = monomeric EYFP; dEYFP = dimeric EYFP. Created with BioRender.com. **(B)** Representative images of U2OS cells expressing EYFP-LacI constructs. Yellow circles signify the largest foci (presumed to be the LacO array). **(C + D)** Quantification of fluorescence intensity **(C)** and area **(D)** at the LacO array. Blue bars indicate monomeric EYFP and green bars represent dimeric EYFP. Error bars show SEM. Statistical analysis was performed using ordinary one-way ANOVA with Dunnett’s multiple-comparison test. Significant differences between TF PrLD constructs and the corresponding LacI-EYFP controls are shown by number signs. ##P < 0.01, ###P < 0.001; ####P < 0.0001. Significant differences between mEYFP and dEYFP constructs are denoted by asterisks. **P < 0.01, ****P < 0.0001. **(E)** Schematic depiction of GFP-Wor1PrLD constructs, which can be TEV treated to remove the MBP tag. **(F + G)** Dimeric Wor1 (dGFP-Wor1PrLD) or monomeric Wor1 (mGFP-Wor1PrLD) constructs were TEV treated at 30°C for 1 h to remove MBP and diluted in 150 mM NaCl, 10 mM Tris-HCl buffer with 5% PEG-8000. Experiment was repeated at least twice. **(F)** Representative images of 20 μM Wor1 protein droplets, imaged immediately after TEV treatment or after aging 3 h at 22°C. **(G)** Wor1 protein samples were diluted to 25 μM and saturation concentrations were determined. Mean concentrations are shown, black circles represent experimental replicates, error bars indicate SEM, and the dotted bar represents starting protein concentration. Statistical analysis was performed using a two-tailed Student’s t-test. *P < 0.05. Scale bar, 10 μm.

To test whether increased valency increases the propensity TFs to undergo liquid-liquid phase separation (LLPS), we examined the phase separation capacity of Wor1 variants expressed in human U2OS cells. U2OS cells contained an array of ~50,000 copies of the LacO sequence inserted into the genome so that LacI-EYFP fusion proteins are recruited to the LacO array (Fig 6A) [16]. Fusion of phase-separating proteins to LacI-EYFP can increase LLPS which, in turn, can increase focal intensity/area at the LacO array (and elsewhere in the nucleus) due to condensate formation [16,43].

We compared the properties of four white-opaque TFs fused to monomeric EYFP (mEYFP) or dimeric EYFP (dEYFP) in U2OS cells. LacI-mEYFP and -dEYFP controls produced comparable foci at the LacO array while the addition of TF PrLDs increased the intensity and/or size of fluorescent puncta at the array (Fig 6B–6D), consistent with PrLD-induced phase separation [43]. Notably, Czf1, Efg1 and Wor1 PrLDs formed brighter and/or larger foci when fused to dEYFP than when fused to mEYFP (Fig 6B–6D). For each PrLD construct, additional foci outside of the LacO array were also generally larger and more abundant fused to dEYFP versus mEYFP (Fig 6B). These results reveal that increasing the valency of the target protein by fusion to dimerizing EYFP versus monomeric EYFP enhances LLPS.

We further investigated the effect of dimerization on Wor1 by purifying recombinant Wor1 PrLD fused to mGFP or dGFP (Fig 6E). A maltose binding protein (MBP) domain was included to increase solubility, which was released by treatment with TEV protease. Both the mGFP- and dGFP-Wor1-PrLD fusion proteins readily formed liquid-like droplets in the presence of the molecular crowding agent PEG-8000 (Fig 6F). Interestingly, no difference in droplet formation or saturation concentration (C_sat_) was observed between the two proteins immediately after removing the MBP tag, but the dimeric construct consistently generated larger assemblies and had a lower C_sat_ after incubation at 22°C for 3 hours (Fig 6F and 6G). These results establish that the Wor1 PrLD fused to dGFP has an increased tendency to self-assemble compared to that fused to mGFP. We therefore propose that the increased white-to-opaque switching observed in Wor1-dGFP cells over Wor1-mGFP cells is due to the increased ability of the dimerized protein to form condensates.

### Rapamycin-induced Wor1 dimerization drives white-to-opaque switching

**Fig 7:**
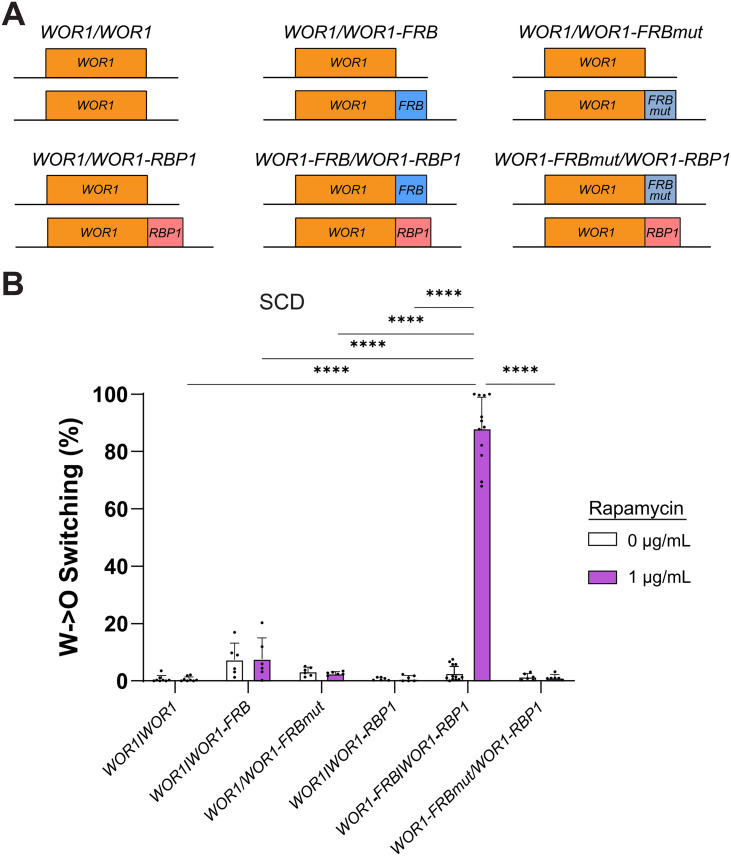
Rapamycin-induced Wor1 dimerization drives white-to-opaque switching. **(A)** Genotypes at the *WOR1* locus for the *C. albicans* strains tested. *WOR1* alleles were tagged with *FRB, *FRBmut** and/or *RBP1* (or left untagged). **(B)** Strains were grown on SCD medium with 1 μg/mL rapamycin or with a vehicle control at 22°C for 7 days. Mean white-to-opaque switching percentages are shown. Black dots indicate biological replicates and error bars show SEM. Statistical analysis was performed using ordinary one-way ANOVA with Dunnett’s multiple-comparison test, in which all mean switching percentages were compared between each other. ****P < 0.0001.

Given that increased Wor1 valency (by fusion to dGFP or by incorporation of extra PrLDs) increases white-to-opaque switching, we tested whether an inducible system of Wor1 dimerization could control switching. Rapamycin is known to induce dimerization between the FK506 binding protein (FKBP) and the FRB domain of the mTOR kinase and has been used as a tool for controlling homo- and hetero-dimerization of protein targets [63–65], and to drive phase separation [66,67]. Here, we fused *RBP1* (the *C. albicans* homolog of *FKBP*) to one *WOR1* allele and *FRB* to the other *WOR1* allele (Fig 7A) in a strain in which *TOR1* was mutated (S1384A) to confer rapamycin resistance [68]. This strain also lacked the native *TOR1* gene and endogenous copies of *RBP1* (so that Wor1-tagged proteins would not interact with native Rbp1). Control strains included those in which *WOR1* was untagged or fused to *FRB* or *RBP1* but not both.

White-to-opaque switching assays were performed on SCD medium (low stochastic switching conditions) in the presence or absence of rapamycin. Control strains expressing *WOR1* fused to *FRB* or *RBP1* exhibited low switching both in the absence and presence of rapamycin (0.7%-7.7%), similar to the untagged control strain (Fig 7B). In contrast, a strain harboring *WOR1-FRB*/*WOR1*-*RBP1* showed low switching in the absence of rapamycin (2.7%) but extremely high switching (88%) when grown in the presence of 1 µg/ml rapamycin (Fig 7B). To further validate that rapamycin-induced Wor1 dimerization was responsible for increased switching, we tested strains with *WOR1* fused to a mutant *FRB* domain (*FRBmut* harboring a S13R mutation) that cannot dimerize with *RBP1*. *WOR1/WOR1-FRBmut* and *WOR1-FRBmut/WOR1-RBP1* strains switched at low frequencies both in the presence and absence of rapamycin (Fig 7B), confirming that dimerization between *WOR1-FRB* and *WOR1-RBP1* mediates increased white-to-opaque switching.

We observed that opaque cells induced by rapamycin in the *WOR1-FRB/WOR1-RBP1* strain were larger and more bulbous than conventional opaque cells (S9A Fig). To confirm that these cells were opaque cells we showed that they expressed the opaque-specific *OP4* gene [69,70] (S9B Fig). We also examined colony phenotypes on CHROMagar plates [56] which showed that rapamycin-induced colonies resembled control opaque colonies (S9C Fig). Together, these results demonstrate that chemical-induced dimerization of *WOR1* efficiently drives cells from white to opaque.

Finally, we also tested whether heterotypic TF-TF interactions could impact white-opaque switching, focusing on those between Wor1 and Wor2 and between Wor1 and Wor4. For these assays, *WOR1* was tagged with *RBP1* and *WOR2* or *WOR4* were tagged with *FRB* (S10A Fig). However, in contrast to the self-dimerization of Wor1 that promoted high levels of white-to-opaque switching, rapamycin-induced hetero-dimerization of Wor1-Wor2 or Wor1-Wor4 did not increase switching (S10B Fig). These data further highlight that Wor1 plays a unique role in driving white-to-opaque switching, and that while Wor1-Wor1 interactions promote switching those between Wor1 and Wor2 or Wor1 and Wor4 do not enhance switching.

## Discussion

The *C. albicans* white-to-opaque switch represents an exemplary system to dissect the transcriptional control of epigenetic cell fate. This switch is controlled by a core network of eight TFs that are potentially recruited to the DNA by their coalescence into biomolecular condensates [36,43,49]. However, despite the identification of >100 TFs whose deletion impacts white-opaque switching [49,58], there remains limited understanding of how these TFs act in combination to regulate the switch. This is in part due to several TFs being essential to switching, with cells lacking *WOR1* or *WOR4* being locked in the white state whereas those lacking *RBF1* or *SSN6* are locked in the opaque state [37,39–42,58].

### Haploinsufficiency analyses establish a unique role for *WOR1*

In this study, we leveraged the diploid nature of *C. albicans* to perform simple and complex haploinsufficiency analyses on core and auxiliary white-opaque TFs to further define their role in phenotypic switching. Consistently, Wor1 was by far the most important TF for switching to the opaque state. For example, in conditions that induced nearly 100% white-to-opaque switching in control strains, *WOR1* heterozygotes underwent only ~0.3% switching whereas the next lowest heterozygote showed ~18% switching. Even the strain that showed the lowest switching frequency of any double heterozygote not involving *WOR1* (a *CZF1/WOR2* heterozygote) showed 44-fold more switching than the *WOR1* single heterozygote.

Haploinsufficiency analyses also revealed that both *SSN6* and *WOR4* heterozygotes showed altered white-opaque switching, even though these heterozygotes were previously reported to switch at wildtype levels [37,58]. The likely reason for this difference is that while *SSN6* and *WOR4* dosage can significantly impact switching frequencies they do so only under a subset of conditions. This highlights how examining switching under different culture conditions can identify factors with modest effects on switching.

### A hyperactive *WOR1* allele enables CHI analyses

**Fig 8:**
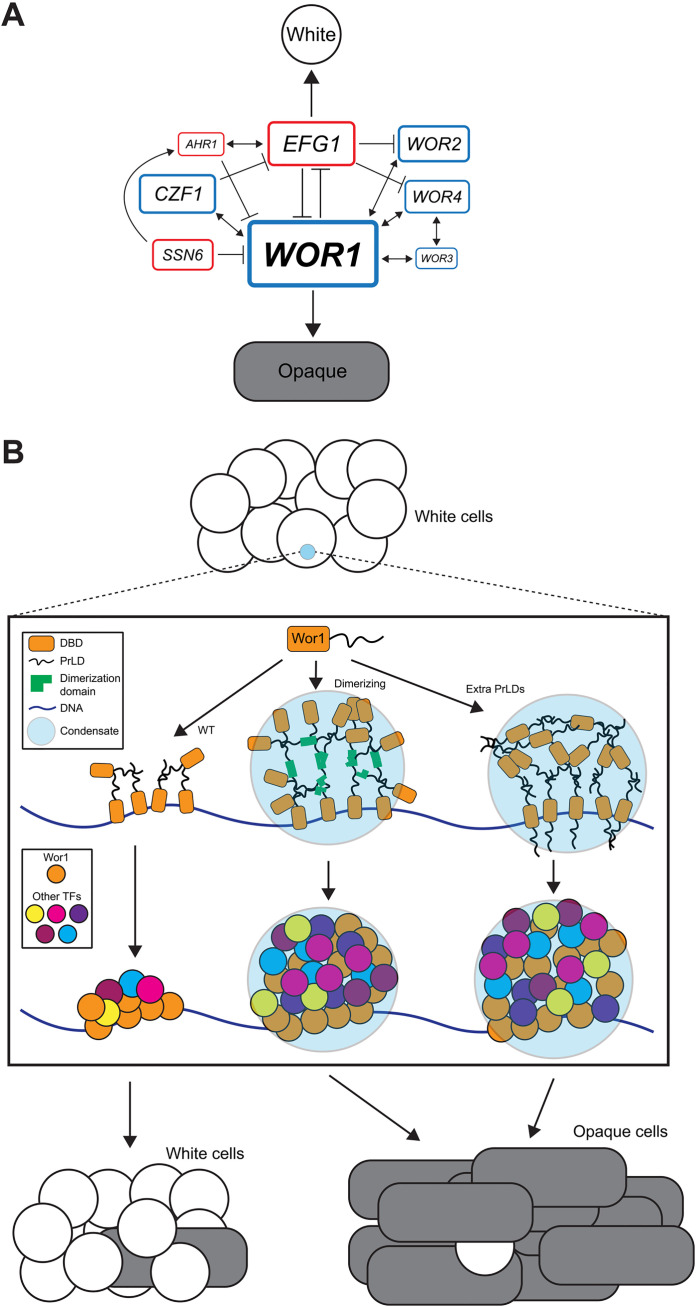
*C. albicans* white-to-opaque switching. **(A)** A core TF network diagram integrating simple and complex haploinsufficiency analyses and prior literature [28]. TFs in blue promote the opaque state and TFs in red promote the white state. Box sizes reflect the size of the TF’s effect on white-opaque switching based on analyses of single TF heterozygotes. One-way arrows indicate positive/buffering interactions, two-way arrows indicate negative/cooperative interactions, and line segments with flat ends indicate repressive interactions between positive and negative regulators. **(B)** Diagram demonstrating how increasing Wor1 valency promotes condensate formation and white-to-opaque switching. Wor1 can recruit other TFs via condensate formation and drive white-to-opaque switching. Wor1 with increased valency (via the addition of extra PrLDs or by forced dimerization) more readily reaches the threshold necessary for condensate formation facilitating TF and transcriptional machinery recruitment and thereby increasing the frequency of white-to-opaque switching.

The relative inability of *WOR1* heterozygotes to undergo white-to-opaque switching led us to utilize strains expressing a hyperactive *WOR1* variant. Substitution of the three positively charged amino acids in the PrLD resulted in a *WOR1*(KR-to-G) variant that increased switching frequencies by more than 30-fold. While the cause of hyperactivity is under investigation, use of this variant allowed us to evaluate *WOR1* heterozygotes for CHI phenotypes with other positive regulators of the switch. These experiments revealed that *WOR1* acts with the core TFs *CZF1*, *WOR2*, *WOR3*, and *WOR4* and with the auxiliary TFs *FLO8*, *GAL4*, and *GZF3* to drive transitions to the opaque state. This is consistent with chromatin immunoprecipitation (ChIP) data showing that several of these TFs co-localize in opaque cells [36,37] and that they can also co-coalesce into condensates [43]. These experiments establish that CHI can dissect genetic interactions between white-opaque TFs (see model in Fig 8A) and is particularly beneficial for examining genes whose homozygous deletion results in an “all-or-nothing” phenotype.

### *WOR1* and white-opaque switching pathways

Previous studies have shown that loss of *WOR1* locks cells in the white state even when other opaque-inducing TFs are ectopically overexpressed [37–40,42,44]. Conversely, ectopic *WOR1* expression can bypass the need for other TFs such as *CZF1*, *WOR2*, and *WOR4* to be present for opaque formation [38,39]. However, despite extensive evidence that *WOR1* is central to opaque formation, it was reported that *wor1*Δ/Δ *efg1*Δ/Δ cells can still switch to the opaque state under strong opaque-inducing conditions (e.g., culture with GlcNAc/CO_2_ at 37°C), indicating that a *WOR1*-independent “alternative opaque pathway” (AOP) exists [71–73]. The AOP was not directly investigated in the current study and opaque cell formation was highly sensitive to *WOR1* dosage under each condition evaluated here. It is possible that the AOP supports opaque formation due to the combinatorial effects of loss of the *EFG1* repressor together with strong opaque-inducing cues, indicating that *WOR1* may be bypassed under these conditions. It was also proposed that *WOR1* promotes opaque formation indirectly via inhibition of *TUP1,* which itself may be recruited to promoters via DNA-binding TFs such as *EFG1* [74]. We found that *EFG1* and *TUP1* gene dosage both altered switching less than *WOR1* dosage, and that *WOR1* dosage impacted switching even in the absence of *EFG1*. Overall, we favor a model whereby *WOR1* promotes opaque cell formation under most conditions, but that opaque cells can also form without Wor1 under certain genetic and environmental conditions like those favoring the AOP.

### Comparison of white-opaque and biofilm TF networks

Biofilm formation contributes directly to *C. albicans* pathogenesis and is regulated by a highly interconnected TF network that shows similarities to the white-opaque network [75,76]. Deletion of single alleles of the biofilm network TFs also results in haploinsufficient phenotypes [51,77], although none exhibited the extreme haploinsufficiency defect of *WOR1* heterozygotes in the white-opaque network. CHI analyses of the biofilm network showed that double TF heterozygotes are often as deficient, if not more so, than homozygous TF deletions [51]. The biofilm network is therefore viewed as a genetically fragile network as it requires multiple TFs to be present at full dosage for efficient biofilm formation to occur [51,77]. In contrast, the white-to-opaque switch is much more dependent on *WOR1* dosage than that of other TFs, and even double TF heterozygotes do not impact switching as much as *WOR1* heterozygotes. The white-opaque network can therefore be considered as a hybrid network that contains a critical node (*WOR1*) whose activity is modulated by a relatively robust network of modifier TFs. This distinguishes the white-opaque network from the more fragile biofilm network that has no central critical node/regulator analogous to *WOR1*.

### The impact of Wor1 valency on white-opaque switching

We were struck by the observation that even a twofold change in *WOR1* dosage could result in a 100-fold change in the white-to-opaque switching frequency. We therefore tested variants with altered valency and found that increased Wor1 valency, either by the addition of extra Wor1 PrLDs or by fusion to dimerizing GFP (dGFP), led to a striking increase in Wor1 function. For example, under conditions where wildtype Wor1 supported 0.1% switching, a strain expressing Wor1 containing an extra PrLD resulted in 11–14% switching while the presence of two extra PrLDs produced close to 100% switching. Similar to Ziv et al. [62], we also found that Wor1 fusion to dGFP resulted in an increase in activity relative to fusion to mGFP (80% versus 12% switching, respectively). In contrast, fusion of dGFP to other network TFs did not substantially alter white-opaque switching frequencies. This demonstrates how Wor1 activity can be enhanced by simple manipulation of its valency.

We also tested whether chemically induced dimerization of Wor1 could be used to control switching. Remarkably, a strain in which *WOR1* alleles were fused to *FRB* and *RBP1* underwent high frequencies of white-to-opaque switching (88% switching) in the presence of rapamycin which induces *FRB*-*RBP1* heterodimerization. However, switching was not induced in this strain in the absence of rapamycin nor in a strain with a mutated FRB that does not undergo dimerization. These results further highlight how changes to TF valency are a powerful approach for manipulating cell state transitions (see Fig 8B) with important implications for the control of cellular reprogramming in higher eukaryotes.

### Connections between TF gene dosage, valency and phase separation

Why do changes in valency and gene dosage result in large-scale changes in Wor1 activity? We propose that this hypersensitivity reflects the impact of these features on the propensity to phase separate. Wor1 and other white-opaque network TFs can form phase-separated condensates due to multivalent interactions mediated by their PrLDs, and mutations that block this ability block opaque cell formation [43]. We found that Wor1 dimerization via fusion to dGFP not only increased its transcriptional activity but also enhanced its phase separation, both in mammalian U2OS cells and when purified and analyzed *in vitro*. We therefore propose that increasing either the valency or the gene dosage of Wor1 increases white-to-opaque switching by increasing the likelihood that this TF reaches the threshold at which it forms phase-separated condensates. These results are in line with other studies that have shown that phase separation events are exquisitely sensitive to both protein valency and expression levels [78–80].

We highlight a recent report in which the products of multiple dosage-sensitive genes were found to encode proteins with the propensity to phase separate [81]. This study emphasized how even small changes in the levels of gene products can alter phase separation events. Indeed, gene dosage sensitivity may represent a more accurate tool for identifying phase-separating factors than current sequence-based predictors [81]. We similarly propose that the extreme dosage sensitivity of *WOR1* reflects the fact that expression levels must reach the threshold at which Wor1 phase separation occurs to drive the white-to-opaque switch. This model aligns with a recent study showing that if Wor1 levels drop below a certain threshold then opaque cells switch back to the white state [82]. Wor1 expression levels are therefore critical for defining both white-to-opaque and opaque-to-white cell transitions.

Finally, the unique role that Wor1 dosage and valency had on white-opaque switching relative to other TFs suggests that this factor may be critical for nucleating the condensates that drive opaque cell formation. Wor1 may also act as a “pioneer factor” to open up condensed chromatin and facilitate the recruitment of other TFs [83]. By analogy, the mammalian pioneer factor FOXA1 can form biomolecular condensates that increase chromatin accessibility, and this process is reliant on the intrinsically disordered regions of FOXA1 for phase separation [84]. Additional studies are now required to test whether *C. albicans* Wor1 acts as a *bona fide* pioneer factor and to further determine how TFs cooperate to control cell fate-determining programs.

## Materials and methods

### Media

*C. albicans* strains were maintained on yeast peptone dextrose (YPD) agar plates, synthetic complete dextrose (SCD) agar plates [85], or CHROMagar plates (Fisher Scientific, NC9514149). Variations of SCD containing different amounts of glucose and N-acetylglucosamine (GlcNAc) were used for switching assays including SCD with 2% glucose, SCD with 1% glucose and 1% GlcNAc, and SCD with 1.75% glucose and 0.25% GlcNAc. All SCD variations used in switching assays were supplemented with 5 μg/mL Phloxine B to stain opaque colonies/sectors on agar plates [86].

### Plasmid construction

Several plasmids were constructed for genomic integration by CRISPR. pADH143 (gift from Aaron Hernday, UC Merced) was amplified by inverse PCR with oligos 4673/4852, gel purified and digested with DpnI. Oligos 6722/6723 were annealed together and inserted into the linearized pADH143 by circular polymerase extension cloning to yield pRB1719. A Golden-Gate-Assembly (GGA)-adapted version of pSFS2A [87] was created by ligation of a BsaI adapter between ApaI/XhoI sites to create pSFS2A-GGA (pRB1397). The adapter was created by annealing oligos 6048/6049 to produce a DNA molecule possessing ApaI and XhoI “sticky” ends. To create a *CaCmWOR1* reintegration plasmid, three pieces were PCR amplified: (1) *CaWOR1* 5’ flank and DBD from genomic DNA (gDNA) with oligos 6726/6727, (2) *CmWOR1* PrLD from pRB1307 with oligos 6730/6731, (3) *CaWOR1* 3’ flank from gDNA with oligos 6728/6729. The three PCR fragments were combined by GGA into pSFS2a-GGA with BsaI to yield pRB1721. To create a *CaCmWOR1*(KR-to-G) reintegration plasmid, three pieces were polymerase chain reaction (PCR) amplified: (1) **C. albicans* WOR1* 5’ flank and DBD from *C. albicans* genomic DNA (gDNA) with oligos 6726/6727, (2) **C. maltosa* WOR1* PrLD KR-to-G mutant from pRB1455 (Gene Universal) with oligos 6730/6731, (3) **C. albicans* WOR1* 3’ flank from gDNA with oligos 6728/6729. The three PCR fragments combined by GGA into pSFS2a-GGA with BsaI to yield pRB1723.

An *OFI1* KO plasmid was created by PCR amplification of the 5’ upstream region of *OFI1* from *C. albicans* SC5314 gDNA with 5811/5812 and the 3’ downstream region with 5813/5814. The 5’ flank and pSFS2a were digested with ApaI/XhoI and ligated together. The 3’ flank was then digested with SacI/SacII and ligated into pSFS2a-5’ flank that was digested with SacI/SacII to yield pRB1569.

A *TUP1* KO plasmid was created via GGA with three pieces: (1) *TUP1* 5’ flank PCR amplified from gDNA with 7018/7019, (2) SatR PCR amplified from pSFS2a with 7022/7023, and (3) *TUP1* 3’ flank PCR amplified from gDNA with 7020/7021. The three PCR components were combined by GGA into pGGA-Select (pRB1754) (NEB) [88] with BsmBI to yield pRB2085.

A monomeric GFP (mGFP) plasmid was created by PCR amplification of dimerizing GFP (dGFP) from pSJS1488 (gift from Steven Sandler, UMass Amherst) into two pieces, split at residue A206, with oligos 8714/8715 and 8716/8717, to mutate the alanine at residue 206 into lysine (A206K). The two PCR products were combined by GGA with BsmBI-HFv2 into pGGA-Select to generate pRB2206. mGFP was amplified from pRB2206 with oligos 8718/8719, digested with XhoI, and ligated into pSFS2a that was digested with XhoI and Antarctic Phosphatase (NEB) treated to generate pSFS2a-mGFP (pRB2219).

To create a monomeric mEYFP, dEYFP was split into two pieces at residue A206 via PCR amplification from a LacI-EYFP plasmid [16] (pRB1208, a gift from Robert Tijan, UC Berkeley) with oligos 8269/8270 and 8271/8272, to mutate the alanine at residue 206 into lysine (A206K). The two EYFP PCR products were assembled by GGA using BsaI-HFv2 into pGGA-Select to generate pRB2060. pRB2060 was then used to generate mEYFP versions of dEYFP-LacI constructs. mEYFP was PCR amplified from pRB2060 with oligos 8275/8276, digested with NheI/BsrGI, and ligated into dEYFP-LacI vectors (pRB1208, pRB1411 and pRB1216 [43]) digested with the same to generate mEYFP-LacI (pRB2108), mEYFP-CmWor1PrLD-LacI (pRB2110), and mEYFP-Czf1PrLD-LacI (pRB2271). The mEYFP-Efg1 variant was generated by PCR amplifying mEYFP from pRB2060 with oligos 5580/8276 and Efg1NPrLD from pRB1222 [43] with oligos 5578/5579. Efg1NPrLD-mEYFP was then generated by fusion PCR joining the two PCR fragments together by PCR with oligos 5578/8276. The Efg1NPrLD-mEYFP product was digested with NheI/BsrGI and ligated into pRB1222 digested with the same to generate Efg1NPrLD-mEYFP-Efg1CPrLD-LacI (pRB2272). The mEYFP-Wor4 variant was created by first PCR amplifying mEYFP from pRB2060 with oligos 5673/8276 and Wor4NPrLD from pRB1266 [43] with oligos 5671/5672. Wor4NPrLD-mEYFP was created by fusion PCR of the two PCR fragments with oligos 5671/8276. The Wor4NPrLD-mEYFP product was digested with NheI/BsrGI and ligated into pRB1266 digested with the same to generate Wor4NPrLD-mEYFP-Wor4CPrLD-LacI (pRB2274).

The *WOR1* 3’ flanking region was PCR amplified from *C. albicans* SC5314 gDNA with oligos 8267/8268, digested with SacI/SacII and ligated into pSFS2a-GGA digested with the same to create pRB2099. Using pRB2099 as a base vector, we created four *WOR1* AB plasmids: #1, WT (DBD-PrLD); #2, PrLD-DBD-PrLD; #3, DBD-PrLD_2_; and #4, PrLD-DBD-PrLD_2_. For #1, the *WOR1* 5’ region and open reading frame (ORF) were PCR amplified from gDNA with oligos 8277/8278 and assembled together by GGA with pRB2099 using BsaI-HFv2 to generate pRB2100. For #2, the *WOR1* 5’ region, *WOR1* PrLD, and full *WOR1* ORF were amplified from gDNA with oligos 8277/8279, 8280/8281, and 8282/8278, respectively. The three PCR products were assembled by GGA with pRB2099 using BsaI-HFv2 to generate pRB2102. For #3, the *WOR1* 5’ region and ORF were amplified from gDNA with oligos 8277/8283 and the *WOR1* PrLD was amplified from gDNA with oligos 8284/8285. The two PCR products were assembled by GGA using pRB2099 and BsaI-HFv2 to generate pRB2106. For #4, four pieces were PCR amplified from gDNA: (1) *WOR1* 5’ region with oligos 8277/8279, (2) *WOR1* PrLD with oligos 8280/8281, (3) *WOR1* ORF with oligos 8282/8283, and (4) *WOR1* PrLD with oligos 8284/8285. The four pieces were assembled by GGA with pRB2099 and BsaI-HFv2 to generate pRB2104.

To create a Wor1-dGFP construct for expression in *E. coli*, we first amplified GFP from pRB719 [43] in two parts using oligos 9529/9530 and 9531/9532, respectively. The two GFP pieces were assembled by GGA into pGGA-Select with BsmBI-V2, yielding pRB2390. pRB2390 was digested with BsiWI-HF and SacI-HF and ligated into pRB719 (digested with the same), yielding MBP-dGFP-Wor1PrLD (pRB2391).

Three plasmids were generated for rapamycin-inducible dimerization of Wor1: pSFS2a-FRB, pSFS2a-FRBmut and pSFS2a-RBP1. *FRB* or *FRBmut* were PCR amplified from *C. albicans* SC5314 WT (RZY47) or TOR1–1 (CAY14787) gDNA, respectively, with oligos 8639/8640, digested with KpnI/ApaI, and ligated into pSFS2a digested with the same to generate pRB2189 and pRB2388, respectively. *RBP1* was PCR amplified from gDNA with oligos 8641/8642, digested with KpnI/ApaI, and ligated into pSFS2a digested with the same to generate pRB2190.

The *OP4*::*mNeonGreen* reporter plasmid was assembled from three PCR products: (1) *OP4* 5’ flanking region amplified from *C. albicans* SC5314 gDNA with oligos 8630/8599, (2) *OP4* 3’ flanking region amplified from gDNA with oligos 8600/8601, and (3) *mNeonGreen*-*SAT1* amplified from pRB895 [69] with oligos 8602/8603. The three PCR products were assembled by GGA using BsaI-HFv2 in pGGA-Select to generate pRB2202.

### *C. albicans* strain construction

Strains heterozygous for core white-opaque network TFs (*AHR1*, *CZF1*, *EFG1*, *SSN6*, *WOR1*, *WOR2*, *WOR3*, *WOR4*) were provided by Alexander Johnson (UCSF) and were *LEU2* auxotrophic. Most double heterozygous *C. albicans* mutants were created using TF deletion plasmids containing the *LEU2* auxotrophic marker flanked by 100–500 bp regions from the 5’ and 3’ homologous regions of each TF (S1 Appendix) [51]. These plasmids were digested with SbfI-HF and used in a lithium-acetate/PEG/heat shock transformation. Double heterozygote mutants created with the *TF*::*LEU2* plasmids are listed in S1 Appendix and were validated by 5’ and 3’ junction check PCRs, with corresponding oligos listed in S1 Appendix. *AHR1* + /Δ *WOR4* + /Δ (CAY13821) and *WOR3* + /Δ *WOR4* + /Δ (CAY13862) double heterozygote mutants were generated by transforming a *WOR4*::*LEU2* construct (PCR amplified from *C. albicans* strain AHY861, a gift from A. Hernday, with oligos 4472/5379) into *AHR1* or *WOR3* single heterozygotes. The double heterozygotes were validated by 5’ and 3’ junction check PCRs with oligos 4472/4835 and 4473/4834. *EFG1* + /Δ *SSN6* + /Δ (CAY13839), *EFG1* + /Δ *WOR3* + /Δ (CAY13844), and *EFG1* + /Δ *WOR4* + /Δ (CAY13846) double heterozygotes were generated by deleting one *EFG1* allele from *SSN6* + /Δ (AHY337 [37]), *WOR3* + /Δ (AHY206, gift from A. Hernday), and *WOR4* + /Δ (MLY1135 [38]) via transforming with ApaI/SacI digested pRB721 [56], and were validated by 5’ and 3’ junction check PCRs with oligos 4438/2284 and 4439/2286. These double heterozygotes were made nourseothricin sensitive by recycling the *SAT1* marker as described [87]. Strains that were auxotrophic for *LEU2* were transformed with a *LEU2* reintegration product (*LEU2* gene that was PCR amplified from *C. albicans* gDNA with oligos 6056/5055), and integration confirmed by PCR with oligos 5058/3965.

The remaining copy of *EFG1* was deleted from an *EFG1* + /Δ strain (AHY119) and from an *EFG1* + /Δ *WOR1* + /Δ strain (CAY14198) by transforming with ApaI/SacI digested pRB721. *EFG1* absence was confirmed with oligos 4479/6548 and validated with 5’ and 3’ junction check PCRs using oligos 7158/4438 and 4439/6984, respectively, to yield *efg1*Δ/Δ (CAY17215) and *efg1*Δ/Δ *WOR1* + /Δ (CAY17218).

*WOR1* heterozygotes were created by removing one copy of *WOR1* from strain RZY47 [42] via transforming with pRB34 digested with KpnI/SacI. *WOR1* heterozygosity was confirmed with 5’ and 3’ PCR junction checks using oligos 1463/4438 and 4439/6672. The presence of either *WOR1* allele A (CAY15051) or *WOR1* allele B (CAY15054) was determined by PCR amplifying *WOR1* ORF using oligos 2390/4239 and digesting with BstEII: allele A (G125, S633) is cut while allele B (A125, N633) is not.

Heterozygotes for auxiliary TFs *ASH1*, *NDT80*, *RLM1*, *RPN4* were created by transforming a *leu2*Δ/Δ strain (AHY120) with Sbf1-HF digested *TF::LEU2* plasmids, creating *ASH1* + /Δ (CAY16732), *NDT80* + /Δ (CAY16734), *RLM1* + /Δ (CAY16737), *RPN4* + /Δ (CAY16739). Junction checks were performed by PCR using oligos listed in S1 Appendix. *TUP1* heterozygotes were created by transforming WT strain AHY135 with the *TUP1* KO plasmid pRB2085 digested with PacI. Deletion of *TUP1* was confirmed by PCR using 4438/6952 and 4439/6953. *SAT1* was then recycled to generate CAY16744.

Additional single and double heterozygous mutants were generated in an SC5314 strain expressing a *WOR1*(KR-to-G) variant which contains the DNA binding domain of *C. albicans WOR1* and the PrLD from *C. maltosa WOR1* in which the K/R residues were mutated to G residues. The *WOR1*(KR-to-G) variant was generated by transforming strain AHY135 via the LEUpOUT CRISPR system [89] with *CmLEU*2 Cas9 (pADH140) together with a *CaWOR1* targeting gRNA (pRB1719) and repair template (pRB1723) to generate strain CAY12264. Transformations were validated by PCR using oligos 6911/6915 and the absence of native *WOR1* was confirmed by PCR using oligos 6911/6914. A *WOR1*(KR-to-G) heterozygote (CAY14328) was generated by deleting one copy of *WOR1* from CAY12264 via transformation with KpnI/SacI-digested pRB34 and recycling the *SAT1* selectable marker, and validated by junction check PCRs with oligos 1393/4438 and 2902/4439. *LEU2* was replaced with a *SAT1* marker in CAY12264 and CAY14328 by transforming these strains with a PCR product amplified from pSFS2a with oligos 8340/8341 and selection on SD Leu- NAT+ plates, and validated by PCR with oligos 4061/4438 and 4439/6057. *SAT1* was recycled to yield SAT-sensitive *WOR1*(KR-to-G) (CAY15131) and *WOR1*(KR-to-G) heterozygote (CAY15133). *TF*::*LEU2* plasmids (digested with Sbf1-HF) were used in transformations with CAY15131 and CAY15133 to create single and double heterozygote mutants (S1 Appendix). Transformants were validated by 5’ and 3’ junction check PCRs, with corresponding oligos listed in S1 Appendix. *WOR3* single and double heterozygotes were generated by PCR amplifying a *WOR3*::*LEU2* deletion cassette from AHY850 (gift from A. Hernday) with oligos 4468/4469 and transforming into CAY15131 and CAY15133 to create *WOR3* + /Δ (CAY15267) and *WOR1* + /Δ *WOR3* + /Δ (CAY15270), and validated by junction check PCRs with oligos 4470/4835 and 4834/4471. *WOR4* single and double heterozygotes were generated by PCR amplifying a *WOR4*::*LEU2* deletion cassette from AHY861 gDNA with oligos 4472/5379 to create *WOR4 + /*Δ (CAY15273) and *WOR1* + /Δ *WOR4* + /Δ (CAY15276), and validated by junction check PCRs with oligos 4472/4835 and 4473/4834. *FLO8* or *OFI1* single and double heterozygotes were generated by transforming strains CAY15131 and CAY15133 with ApaI/SacI-digested pRB989 [90] or pRB1569 plasmids to delete one copy of *FLO8* or *OFI1*, respectively. *FLO8* and *OFI1* heterozygotes were validated by junction check PCRs with oligos 6425/4438 + 4439/6426, and 7182/4438 + 4439/7181, respectively. *SAT1* was recycled and *LEU2* was added back by transforming with DNA PCR amplified from *C. albicans* gDNA with oligos 6056/5055, and junction checked by PCR with 5058/3965 to yield the following strains: *FLO8* + /Δ (CAY15177), *WOR1* + /Δ *FLO8* + /Δ (CAY15180), *OFI1* + /Δ (CAY15222), and *WOR1* + /Δ *OFI1* + /Δ (CAY15225). *TUP1* heterozygotes were created by transforming CAY15131 and CAY15133 with the *TUP1* KO plasmid (pRB2085) digested with PacI. Deletion of *TUP1* was confirmed by PCR using oligos 4438/6952 and 4439/6953. *SAT1* was recycled generating *TUP1* + /Δ (CAY16750), and *WOR1* + /Δ *TUP1* + /Δ (CAY16755).

To generate GFP-tagged TF strains, mGFP and dGFP were PCR amplified from pRB2219 and pADH76 [38], respectively, and used to transform RZY47. *AHR1-GFP* constructs were PCR amplified with oligos 8779/8880 to generate *AHR1/AHR1-mGFP* (CAY15336) and *AHR1/AHR1-dGFP* (CAY15361), validated by junction check PCRs with oligos 4902/8817 and 4439/4903. The second *AHR1* allele was GFP-tagged and checked by PCR with oligos 4439/4903, and oligos 4902/4903 + 3814/4903 were used to confirm both alleles were tagged, generating *AHR1-mGFP/AHR1-mGFP* (CAY15471) and *AHR1-dGFP/AHR1-dGFP* (CAY15468). *CZF1-GFP* constructs were PCR amplified with oligos 8777/8778 and used to generate *CZF1/CZF1-mGFP* (CAY15340) and *CZF1/CZF1-dGFP* (CAY15338), validated by junction check PCRs with oligos 3804/3925 and 4439/4899. The second *CZF1* allele was GFP-tagged and checked by PCR with oligos 4439/4899, and oligos 5477/4899 and 3814/4899 were used to confirm both alleles were tagged, generating *CZF1-mGFP/CZF1-mGFP* (CAY15629) and *CZF1-dGFP/CZF1-dGFP* (CAY15642). *EFG1-GFP* tags were PCR amplified with oligos 8773/8774 to generate *EFG1/EFG1-mGFP* (CAY15446) and *EFG1/EFG1-dGFP* (CAY15464), validated by junction check PCRs with oligos 4479/8817 and 4439/7157. The second *EFG1* allele was GFP-tagged and checked by PCR with oligos 4439/6984, and oligos 4561/6984 + 3814/6984 were used to confirm both alleles were tagged, generating *EFG1-mGFP/EFG1-mGFP* (CAY15560) and *EFG1-dGFP/EFG1-dGFP* (CAY15558). *SSN6-GFP* constructs were PCR amplified with oligos 8809/8810 to generate *SSN6/SSN6-mGFP* (CAY15449) and *SSN6/SSN6-dGFP* (CAY15447), validated by junction check PCRs with oligos 5693/8817 and 4439/7191. The second *SSN6* allele was GFP-tagged and checked by PCR with oligos 4439/5684, and oligos 5693/5684 and 162/5684 were used to confirm both alleles were tagged, generating *SSN6-mGFP/SSN6-mGFP* (CAY15608) and *SSN6-dGFP/SSN6-dGFP* (CAY15605). *WOR1-GFP* tags were amplified with oligos 8771/8772 to generate *WOR1/WOR1-mGFP* (CAY15359) and *WOR1/WOR1-dGFP* (CAY15334), validated by junction check PCRs with oligos 25/8871 and 4439/6916. The second *WOR1* allele was GFP-tagged and checked by PCR with oligos 4439/6916, and oligos 25/3925 and 25/6916 were used to confirm both alleles were tagged, generating *WOR1-mGFP/WOR1-mGFP* (CAY15466) and *WOR1-dGFP/WOR1-dGFP* (CAY15346). *WOR2-GFP* tags were PCR amplified with oligos 8811/8812 to generate *WOR2/WOR2-mGFP* (CAY15453) and *WOR2/WOR2-dGFP* (CAY15451), validated by junction check PCRs with oligos 3801/8817 and 4439/4889. The second *WOR2* allele was GFP-tagged and checked by PCR with oligos 4439/4889, and oligos 3801/4889 and 3814/4889 were used to confirm both alleles were tagged, generating *WOR2-mGFP/WOR2-mGFP* (CAY15477) and *WOR2-dGFP/WOR2-dGFP* (CAY15474). *WOR3-GFP* constructs were PCR amplified with oligos 8813/8814 and used to generate *WOR3/WOR3-mGFP* (CAY15457) and *WOR3/WOR3-dGFP* (CAY15455), validated by junction check PCRs with oligos 3944/8817 and 4439/4469. The second *WOR3* allele was GFP-tagged and checked by PCR with oligos 4439/4469, and oligos 3944/4469 and 3814/4469 were used to confirm both alleles were tagged, generating *WOR3-mGFP/WOR3-mGFP* (CAY15564) and *WOR3-dGFP/WOR3-dGFP* (CAY15562). *WOR4-GFP* tags were PCR amplified with oligos 8815/8816 and used to generate *WOR4/WOR4-mGFP* (CAY15461) and *WOR4/WOR4-dGFP* (CAY15459), validated by junction check PCRs with oligos 3803/8817 and 4439/4473. The second *WOR4* allele was GFP-tagged and checked by PCR with oligos 4439/4473, and oligos 3803/4473 and 3814/4473 were used to confirm both alleles were tagged, generating *WOR4-mGFP/WOR4-mGFP* (CAY15610) and *WOR4-dGFP/WOR4-dGFP* (CAY15631).

Efg1-GFP Wor1-GFP strains were generated by PCR amplifying mGFP-SatR and dGFP-SatR tags for *WOR1* from pRB2219 and pADH76, respectively, with oligos 8771/8772. CAY15446 was transformed to tag *WOR1* with mGFP and CAY15464 was transformed to tag *WOR1* with dGFP. Junction checks were performed by PCR with oligos 25/8817 and 4439/6916. *SAT1* was recycled yielding *EFG1-mGFP WOR1-mGFP* (CAY16791) and *EFG1-dGFP WOR1-dGFP* (CAY16794).

Strains with altered Wor1 valency were generated by integrating the four *WOR1* addback constructs (#1 – pRB2100, #2 – pRB2102, #3 – pRB2106, or #4 – pRB2104) digested with ApaI/SacI into *wor1*Δ*/*Δ strain CAY2019. Correct integration of each construct was checked by PCR with oligos 1393/4438 and 4439/6672. *SAT1* was recycled from each strain to yield: *WOR1* AB #1 (CAY14499), *WOR1PrLD-WOR1* AB #2 (CAY14502), *WOR1-WOR1PrLD* AB #3 (CAY14507), and *WOR1PrLD-WOR1-WOR1PrLD* AB #4 (CAY14505). *WOR1* was C-terminally tagged with mNeonGreen in all four constructs by transforming with PCR cassettes amplified from pRB2174 with oligos 8439/8440 (constructs #1 and #2) or 9208/8440 (constructs #3 and #4) to yield *WOR1-mNeonGreen* (CAY16428), *WOR1PrLD-WOR1-mNeonGreen* (CAY16430), *WOR1-WOR1PrLD-mNeonGreen* (CAY16432), and *WOR1PrLD-WOR1-WOR1PrLD-mNeonGreen* (CAY16434). Transformations were checked by PCR using oligos 5018/7594 and 9075/6916.

The rapamycin-inducible Wor1 dimerization strains were created in a *TOR1–1* (*TOR1* S1972A) mutant (JRB212, gift from Joseph Heitman, Duke University). Both copies of *RBP1* were removed from JRB212 via transformations with PCR cassettes amplified from pSFS2a with oligos 8286/8287. Transformations were checked by PCR with oligos 8332/8333 and *SAT1* was recycled to generate CAY14483. *TOR1* was removed from CAY14483 by transforming with a cassette PCR amplified from pSFS2a with oligos 8334/8335 and integrants were checked by PCR with oligos 4439/8337. The resulting mutant retained the *TOR1–1* allele (and not the WT *TOR1* allele), confirmed by PCR on the strain with oligos 8533/8534 and digestion with NheI, which only cuts the wildtype allele. *SAT1* was recycled to generate CAY14699, which was transformed with ApaI/SacI digested pRB727 to remove *MTLα*, verified by PCR with oligos 1/2 and 3/4. *SAT1* was recycled to generate CAY14787. *FRB-SAT1* and *FRBmut-SAT1* were PCR amplified from pRB2189 and pRB2388, respectively, with oligos 8710/8437 to fuse with the *WOR1* ORF in the switching competent *rbp1*Δ*/*Δ *TOR1–1* + /Δ strain, verified by junction check PCR with oligos 8694/6911 and 4439/6672. *SAT1* was recycled to generate *WOR1/WOR1-FRB* (CAY14614) and *WOR1/WOR1-FRBmut* (CAY16853). *RBP1-SAT1* was PCR amplified from pRB2190 with oligos 8711/8437 and transformed into CAY14787 (verified by PCR checks with oligos 8696/6911 and 4439/6672) and into CAY14614 (verified by PCR checks with oligos 25/4438 and 4439/6672, while PCR with oligos 25/8694, 8695/6672, and 25/6916 were used to confirm that both alleles were tagged). *SAT1* was recycled to generate strains *WOR1/WOR1-RBP1* (CAY14620) and *WOR1-FRB/WOR1-RBP1* (CAY14625). *FRBmut-SAT1* was PCR amplified from pRB2388 with oligos 8710/8437 to fuse with the *WOR1* ORF in CAY16420, verified by junction check PCRs with oligos 25/4438 and 4439/6672, while PCR with oligos 25/8694, 8695/6672, and 25/6916 were used to confirm that both alleles were tagged. *SAT1* was recycled to generate *WOR1-FRBmut/WOR1-RBP1* (CAY16856). CAY14625 was transformed with PacI-digested pRB2202 to incorporate the *OP4*::*mNeonGreen* reporter and verified by PCR with oligos 1389/5823 and 4439/6279, creating CAY14781.

The second *WOR1* allele was fused with *RBP1* by transforming CAY14620 with a PCR cassette amplified from pRB2190 with oligos 8711/8437. Integration was confirmed by PCR checks with oligos 25/4438 and 4439/6916; the first allele was still tagged by PCR checks with oligos 25/8696 and 8697/6916. *SAT1* was recycled generating *WOR1-RBP1/WOR1-RBP1* (CAY15820). Using two rounds of transformations starting with CAY14787 and CAY15820, respectively, *WOR2-FRB/WOR2-FRB* and *WOR1-RBP1/WOR1-RBP1 WOR2-FRB/WOR2-FRB* were created by transforming with *FRB* cassettes PCR amplified from pRB2189 with 8984/8812. The first *WOR2-FRB* allele was checked by PCR with oligos 8693/4889 and the second *WOR2-FRB* allele was checked by PCR with oligos 4439/4889. *SAT1* was recycled generating *WOR2-FRB* (CAY16089) and *WOR1-RBP1 WOR2-FRB* (CAY16103). Using two rounds of transformations starting with CAY14787 and CAY15820, respectively, *WOR4-FRB/WOR4-FRB* and *WOR1-RBP1/WOR1-RBP1 WOR4-FRB/WOR4-FRB* were created by transforming with FRB cassettes PCR amplified from pRB2189 with oligos 8986/8816. The first *WOR4-FRB* allele was checked by PCR with oligos 8693/4473 and the second *WOR4-FRB* allele was checked by PCR with oligos 4439/4473. *SAT1* was recycled generating *WOR4-FRB* (CAY16093) and *WOR1-RBP1 WOR4-FRB* (CAY16106).

### White-opaque switching assays

For white-to-opaque switching assays, *C. albicans* strains were cultured on YPD agar plates at 30°C to isolate single colonies 1–7 d prior to the experiment. SCD or derivative plates were poured 1–4 d prior to the experiment. *C. albicans* colonies in the white state were collected, diluted in H_2_O, plated for ~100 colonies per plate, and incubated for 7 d. After the 7-day incubation, white-to-opaque switching frequencies were scored by dividing the number of opaque colonies (whole opaque colonies or white colonies with opaque sectors) by the total colony number. Each biological replicate was performed with at least two technical replicates, each of which had 50–130 colonies. For rapamycin-induced switching assays, SCD plates were supplemented with either 1 μg/mL rapamycin in ethanol or with ethanol alone as a vehicle control (1 mL 100% ethanol with or without rapamycin per 1 L SCD). Opaque-to-white assays were performed using the same methods except starting with opaque cells instead of white cells.

For complex haploinsufficiency (CHI) assays, switching frequencies in TF heterozygotes were normalized to those in WT cells. As white-opaque switching frequencies varied from experiment to experiment, biological replicates were normalized to WT controls analyzed on the same day. Normalized switching frequencies from single heterozygous *C. albicans* mutants were multiplied together to determine expected switching frequencies of double heterozygous mutants.

### Gene expression by qRT-PCR

*C. albicans* strains were grown on SCD plates before overnight culture in liquid SCD at 22°C. Cultures were diluted 1:20 in fresh SCD medium and grown for 5.5 h at 22°C to reach mid-log phase before RNA was harvested. Cell state (white or opaque) was confirmed by microscopy. Opaque *WOR1* + /Δ heterozygotes were unstable in SCD at 22°C so RNA was harvested from WT opaque and *WOR1* heterozygote opaque colonies grown for 5 d on SCD + 1% GlcNAc plates at 22°C which supported a stable opaque state. RNA was harvested with the MasterPure Yeast RNA Purification Kit (BioSearch). RNA samples were DNAse I treated and reverse transcribed into cDNA with the iScript Reverse Transcription kit (Bio-Rad). cDNA was used at a final dilution of 1:100 with iTaq Universal SYBR Green Supermix (Bio-Rad) for qRT-PCR. Oligos used for qRT-PCR are described in S1 Appendix. qRT-PCR reactions were performed with the following conditions: 95°C for 3 min, [95°C for 30 s, 54.2°C for 30 s, 60°C for 30 s] for 49 cycles, 60°C for 10 min on a CFX384 Touch Real-Time PCR Detection System (BioRad). qRT-PCR data analysis was performed with the ΔΔ CT method, using *ACT1* as a reference gene and normalized to WT strains for each gene analyzed. Three technical replicates for each biological replicate were used for each gene analyzed.

### Yeast cell imaging and fluorescence quantification

*C*. *albicans* strains were grown overnight in SCD medium at 22°C. Overnight cultures were diluted 1:5 in fresh medium and Hoescht 33258 was added to a final concentration of 1 μg/mL. Cultures were rotated at 22°C for 20 min, washed in fresh medium, and made into wet mount slides. Cells were imaged on a Zeiss Axio Observer Z1 inverted fluorescence microscope at 63X magnification together with a 1.6X OptoVar. The microscope was equipped with AxioVision (v.4.8) and Zen software (v.3.0 blue edition). Post-imaging processing was carried out in FIJI (ImageJ v.2.9.0).

To quantify TF expression, cell nuclei were outlined using the Hoescht stain in FIJI, overlayed on the GFP channel, and analyzed for mean fluorescence intensity. Background fluorescence was corrected for by measuring the fluorescence in an area without cells and subtracting it from the nuclear fluorescence intensity. At least 10 cells were imaged per experiment, and experiments were conducted at least in duplicate.

### Mammalian cell culture, live cell imaging, and LacO array analysis

A U2OS (human sarcoma) cell line engineered to contain ~50,000 copies of the LacO array was used [16]. U2OS cells were maintained at 37°C with 5% CO_2_ in Dulbecco’s modified Eagle’s medium (DMEM, Thermo Fisher Scientific) supplemented with 10% heat-inactivated fetal bovine serum (FBS, Thermo Fisher Scientific) and 1% penicillin-streptomycin (Thermo Fisher Scientific). To transfect constructs, U2OS cells were seeded onto 24-well glass bottom plates (Cellvis). The following day, DMEM + FBS + 1% pen/strep was replaced with DMEM without additives. LacI plasmid constructs were added to wells with Lipofectamine 3000, P3000, and OptiMEM (Thermo Fisher Scientific) according to the manufacturer instructions. After 4 h, DMEM + FBS + 1% pen/strep replaced the DMEM without additives. Transfected U2OS cells were incubated overnight at 37°C with 5% CO_2_ and imaged with an APEXVIEW APX100 inverted fluorescence microscope equipped with a Hammamatsu Orca-Fusion CMOS camera and gradient contrast at 40X magnification. The microscope was equipped with cellSens software (V4.1).

Image analysis was performed in FIJI (ImageJ v.2.9.0). A perimeter was drawn around the LacO array (the brightest focus) inside the U2OS nucleus and was analyzed for mean fluorescence intensity and area. Mean fluorescence intensity was corrected for by subtracting the intensity from a spot immediately outside the array. At least 20 cells were imaged per experiment, and experiments were conducted at least in duplicate.

### Protein purification

Protein expression constructs were transformed into BL21 (DE3) Star *E*. *coli* cells. Cells were grown overnight at 37°C in Luria broth (LB) medium, diluted 1:100 in fresh LB, grown at 37°C to an OD_600_ of 0.7-0.9, and induced with 1 mM isopropyl β-D-1-thiogalactopyranoside at 25°C for 4 h. Cells were lysed with lysozyme and sonicated in lysis buffer (10 mM Tris, pH 7.4, 1 M NaCl, 10 mM imidazole, and a protease inhibitor cocktail; Thermo Scientific Pierce Protease Inhibitor). Wor1 protein was purified by nickel affinity column chromatography, followed by size exclusion column chromatography on a Sephacryl S300 26/60 column (GE Healthcare). Protein fractions were collected and concentrated in Amicon Ultra 50K concentrators (Millipore), then frozen in liquid nitrogen and stored at -80°C.

### Phase separation assays

Proteins were thawed at 22°C and diluted into 10 mM Tris-HCl, pH 7.4, 150 mM NaCl buffer. Proteins were concentrated in Amicon Ultra 0.5-ml centrifugal filter units (Millipore) and concentrations determined using a Nanodrop 2000c (Thermo Fisher Scientific). Extinction coefficients were predicted from sequence compositions using ProtParam (Expasy). Samples were further diluted in 10 mM Tris-HCl, pH 7.4, 150 mM NaCl to the appropriate concentration for each assay. Samples were TEV treated with 1:10 ratio TEV (0.3 mg/ml) to protein and incubated for 1 h at 30°C. 5% PEG-8000 was included as a molecular crowding agent. Following TEV treatment, proteins were either imaged or centrifuged to quantify phase separation. For droplet imaging, Wor1 constructs were imaged in 10-well chamber slides (Polysciences) with 2 μl protein solution per well sealed under a glass coverslip immediately or after aging for the indicated time at 22°C. Images were acquired with a Zeiss Axio Observer Z1 inverted fluorescence microscope for fluorescence and DIC imaging at 63x magnification. Post-imaging processing was carried out in FIJI (ImageJ v.2.9.0).

To quantify Wor1 phase separation, absorbance at 280 nm (A_280_) was first measured to calculate total protein concentration. Protein solutions were then centrifuged at 14,000*g* for 10 min to separate phase-separated protein from soluble protein. The A_280_ of the supernatant was measured to calculate soluble protein. The A_280_ corresponding to TEV protease was also measured and subtracted from the measured A_280_ values. Phase separated protein was calculated by subtracting the supernatant concentration from the total concentration. Finally, the saturation concentration was calculated by subtracting the phase separation concentration from the total starting protein concentration.

## Supporting information

S1 Fig Comparison of *WOR1* A and B alleles in white-to-opaque switching. **(A)** Allelic differences in the *WOR1* ORF. Allele A: G125, S633; allele B: A125, N633. **(B)** Strains were grown on SCD + 1% GlcNAc in the presence of 10% CO_2_ for 24 h before outgrowth in normoxia. Switching frequencies were determined after growth at 22°C for 7 days. Black dots indicate biological replicates and error bars show SEM. Statistical analysis was performed using ordinary one-way ANOVA with Dunnett’s multiple-comparison test, in which all switching percentages were compared to each other. ***P < 0.001; ****P < 0.0001.
(S1_Fig.TIF)

S2 Fig Gene expression of network white-opaque TFs in heterozygotes. **(A-C)** WT and TF heterozygote strains were grown to mid-log phase in liquid SCD before RNA was harvested for qRT-PCR analysis. Mean normalized expression is shown. Black dots indicate biological replicates, error bars show SEM, and the dotted line corresponds to gene expression in the WT control. **(A)** TF gene expression from corresponding TF single heterozygotes is expressed relative to *ACT1* and normalized to WT in both white (left) and opaque (right) cell types. Statistical analysis was performed using a two-tailed Student’s t-test. *P < 0.05. **(B)** TF gene expression from WT white cells is expressed relative to *ACT1* and normalized to the WT opaque cells. Statistical analysis was performed using a two-tailed Student’s t-test, in which the fold expression was compared between the white and opaque cell states. *P < 0.05; **P < 0.01. **(C)** *WOR1*, *WOR3*, and *WOR4* gene expression is shown (relative to *ACT1* and normalized to WT opaque) in opaque *WOR3* and *WOR4* single heterozygotes and opaque *WOR3 WOR4* double heterozygotes.
(S2_Fig.TIF)

S3 Fig *CaWOR1* vs. *CaCmWOR1* constructs in white-to-opaque switching. **(A)** Genotypic differences between *CaWOR1*, *CaCmWOR1*, and *CaCmWOR1(KR-to-G)*. All three constructs contain the *C. albicans WOR1* DNA binding domain (DBD; orange). *CaWOR1* contains the *C. albicans WOR1* PrLD (blue) while *CaCmWOR1* constructs contain the *C. maltosa WOR1* PrLD (light cyan). **(B)** All three constructs were grown on SCD (white) or SCD supplemented with 1% GlcNAc (gray) and switching frequencies determined after growth at 22°C for 7 days. Black dots indicate biological replicates and error bars show SEM. Statistical analysis was performed using ordinary one-way ANOVA with Dunnett’s multiple-comparison test, in which normalized switching frequencies were compared between each strain. ***P < 0.001; ****P < 0.0001; ns = not significant.
(S3_Fig.TIF)

S4 Fig White-to-opaque switching frequencies of auxiliary TF heterozygotes. Strains were grown on SCD + 1% GlcNAc + 16 h 10% CO_2_ before outgrowth on normoxia. Switching frequencies were determined after growth at 25°C for 7 days. Mean white-to-opaque switching percentages are shown on the left y-axis and mean normalized white-to-opaque switching frequencies are shown on the right y-axis relative to the TF + / + control from same-day experiments. Black dots indicate biological replicates and error bars show SEM. Statistical analysis was performed using ordinary one-way ANOVA with Dunnett’s multiple-comparison test, in which heterozygote switching frequencies were compared to the WT strain. ****P < 0.0001.
(S4_Fig.TIF)

S5 Fig White-to-opaque switching frequencies of double TF heterozygotes in a *WOR1*(KR-to-G) strain background. **(A)** Genotypes of the strains used. **(B)** WT, single heterozygotes and double heterozygotes in the *WOR1*(KR-to-G) strain background were grown on SCD + 1% GlcNAc in 10% CO_2_ for 20 h before outgrowth under normoxia. Switching frequencies were determined after growth at 25°C for 7 days. Mean white-to-opaque switching percentages are shown on the left y-axis and mean normalized white-to-opaque switching frequencies are shown on the right y-axis relative to WT from same-day experiments. Black dots indicate biological replicates and error bars show SEM. *WOR1* single heterozygotes and double heterozygotes are shown in white; WT and TF single heterozygotes are shown in gray. Statistical analysis was performed using ordinary one-way ANOVA with Dunnett’s multiple-comparison test, in which switching frequencies were compared between each strain. *P < 0.05; **P < 0.01; ***P < 0.001; ****P < 0.0001.
(S5_Fig.TIF)

S6 Fig Switching assays investigating *EFG1* and *WOR1* epistasis. **(A)** WT, single, and double heterozygote strains in the *WOR1*(KR-to-G) background were grown on SCD. **(B)** WT, single, and double heterozygote strains in the *WOR1*(WT) background were grown on SCD + 1% GlcNAc + 20 h 10% CO_2_ before outgrowth in normoxia. **(C)** WT and *WOR1* + /Δ (white cells) or *efg1*Δ/Δ and *efg1*Δ/Δ*WOR1* + /Δ (gray cells) were grown on SCD. In **A-C**, switching frequencies were determined after growth at 25°C for 7 days. For **A** and **B**, mean white-to-opaque switching percentages are shown on the left y-axis and mean normalized white-to-opaque switching frequencies are shown on the right y-axis relative to the WT control from same-day experiments. For **C**, mean percentages of switching to the opaque state are shown on the left y-axis. Black dots indicate biological replicates and error bars show SEM. Statistical analysis was performed using ordinary one-way ANOVA with Dunnett’s multiple-comparison test, in which switching frequencies of each strain were compared to each other. ****P < 0.0001.
(S6_Fig.TIF)

S7 Fig Wor1-mNeonGreen construct expression and white-to-opaque switching. **(A)** Wor1 PLD variants were C-terminal tagged with mNeonGreen. **(B)** Wor1-mNeonGreen levels were quantified in opaque cells. Mean mNeonGreen expression levels are shown with error bars representing SEM. Statistical analysis was performed using ordinary one-way ANOVA with Dunnett’s multiple-comparison test, in which normalized switching frequencies were compared between each strain. *P < 0.05; **P < 0.01; ***P < 0.001; ****P < 0.0001. **(C)** Representative images of untagged or mNeonGreen-tagged opaque cells. Scale bar, 10 μm. **(D)** Strains were grown on SCD (left) or SCD + 48 h 5% CO_2_ before outgrowth in normoxia (right). Switching frequencies were determined after growth at 25°C for 7 days. Mean white-to-opaque switching percentages are shown. Black dots indicate biological replicates and error bars show SEM. Statistical analysis was performed using ordinary one-way ANOVA with Dunnett’s multiple-comparison test, in which switching frequencies of each strain were compared to each other. *P < 0.05; **P < 0.01; ****P < 0.0001.
(S7_Fig.TIF)

S8 Fig TF-GFP expression in white and opaque cells. **(A)** TF-GFP levels were quantified in white and opaque cells. Cells were untagged (white bars) or tagged with mGFP (light cyan bars) or with dGFP (light green bars). Mean GFP expression levels are shown with error bars representing SEM, and the dotted line corresponding to the untagged control. Statistical analysis was performed using a two-tailed Student’s t-test in which mean GFP expression was compared between mGFP and dGFP for each TF. ****P < 0.0001. **(B)** Representative images of untagged cells or cells with both alleles of a TF tagged with mGFP or dGFP. Images are representative of two independent experimental replicates. Scale bar, 10 μm.
(S8_Fig.TIF)

S9 Fig Rapamycin-induced opaque cells. **(A)** Opaque cells from *C. albicans WOR1-FRB/WOR1-RBP1* strain imaged in DIC. Scale bar, 10 μm. **(B)** White colonies with opaque sectors (denoted by red arrows) and corresponding white and opaque cells from a *WOR1-FRB/WOR1-RBP1* strain with an *OP4::mNeonGreen* reporter. Scale bar, 10 μm. **(C)** *WOR1-FRB/WOR1-RBP1* opaque and white cells cultured on CHROMagar at 22°C for six days.
(S9_Fig.TIF)

S10 Fig White-to-opaque switching in strains harboring rapamycin-inducible TF-TF dimerization constructs. **(A)** Genotypes at *WOR1*, *WOR2*, and *WOR4* loci in the six *C. albicans* strains tested. **(B)** Strains were grown on SCD medium at 22°C with 1 μg/mL rapamycin or with a vehicle control for 7 days. Mean white-to-opaque switching percentages are shown; black dots indicate biological replicates, and error bars show SEM.
(S10_Fig.TIF)

S1 Appendix Plasmids, oligonucleotides, and strains used in this study. (S1_Appendix.XLSX)

S2 Appendix Experimental data for each figure in this study. (S2_Appendix.XLSX)
