# Supplementary figures and images for: Gene dosage and protein valency impact phase separation and fungal cell fate
Source: PLoS Genet. 2025 Aug 8;21(8):e1011810. doi: 10.1371/journal.pgen.1011810 (PMC12333994; doi:10.1371/journal.pgen.1011810)

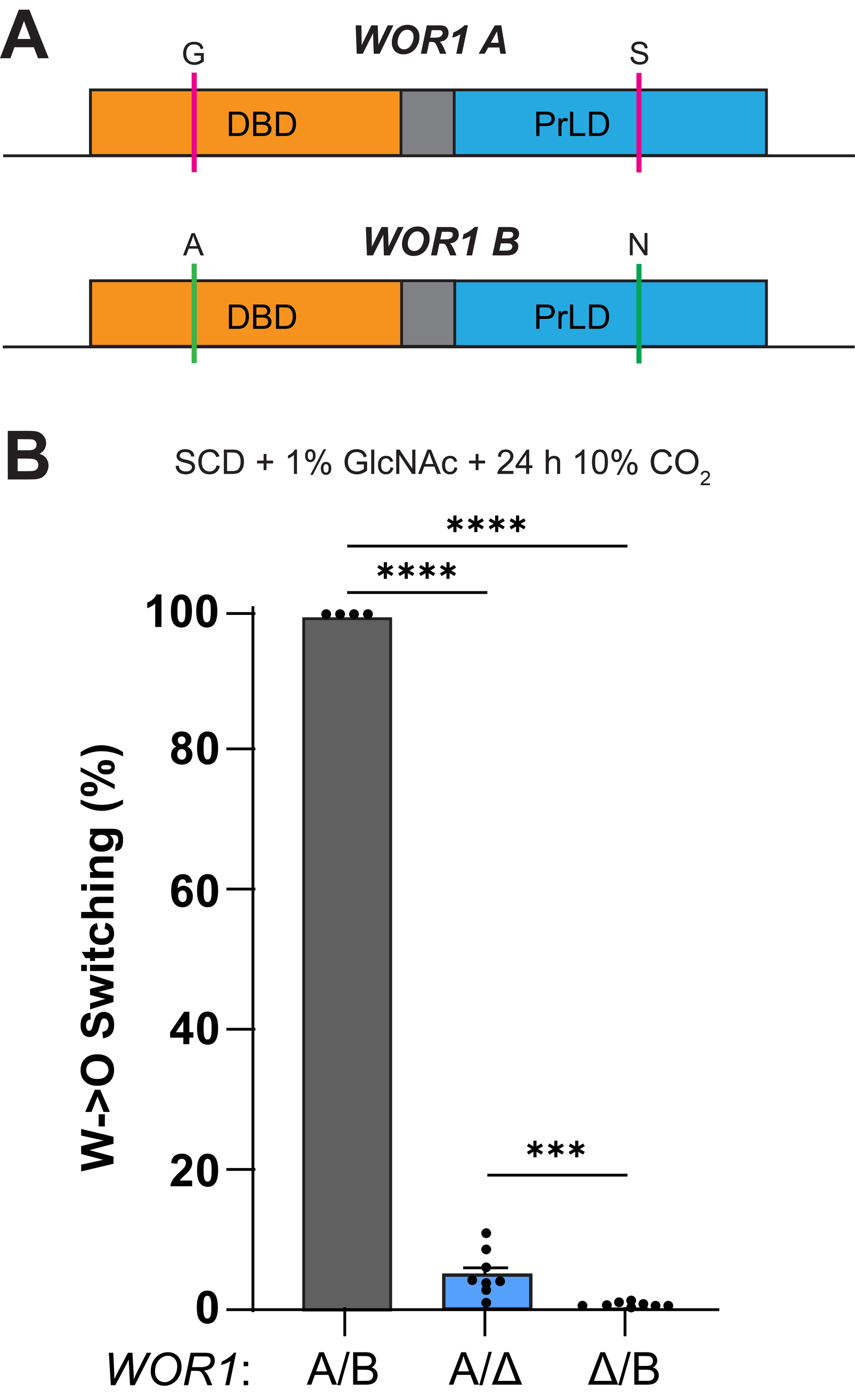

Supplement: S1 Fig — (A) Allelic differences in the WOR1 ORF. Allele A: G125, S633; allele B: A125, N633. (B) Strains were grown on SCD + 1% GlcNAc in the presence of 10% CO2 for 24 h before outgrowth in normoxia. Switching frequencies were determined after growth at 22°C for 7 days. Black dots indicate biological replicates and error bars show SEM. Statistical analysis was performed using ordinary one-way ANOVA with Dunnett’s multiple-comparison test, in which all switching percentages were compared to each other. ***P < 0.001; ****P < 0.0001. (S1_Fig.TIF) [file pgen.1011810.s001.tif]

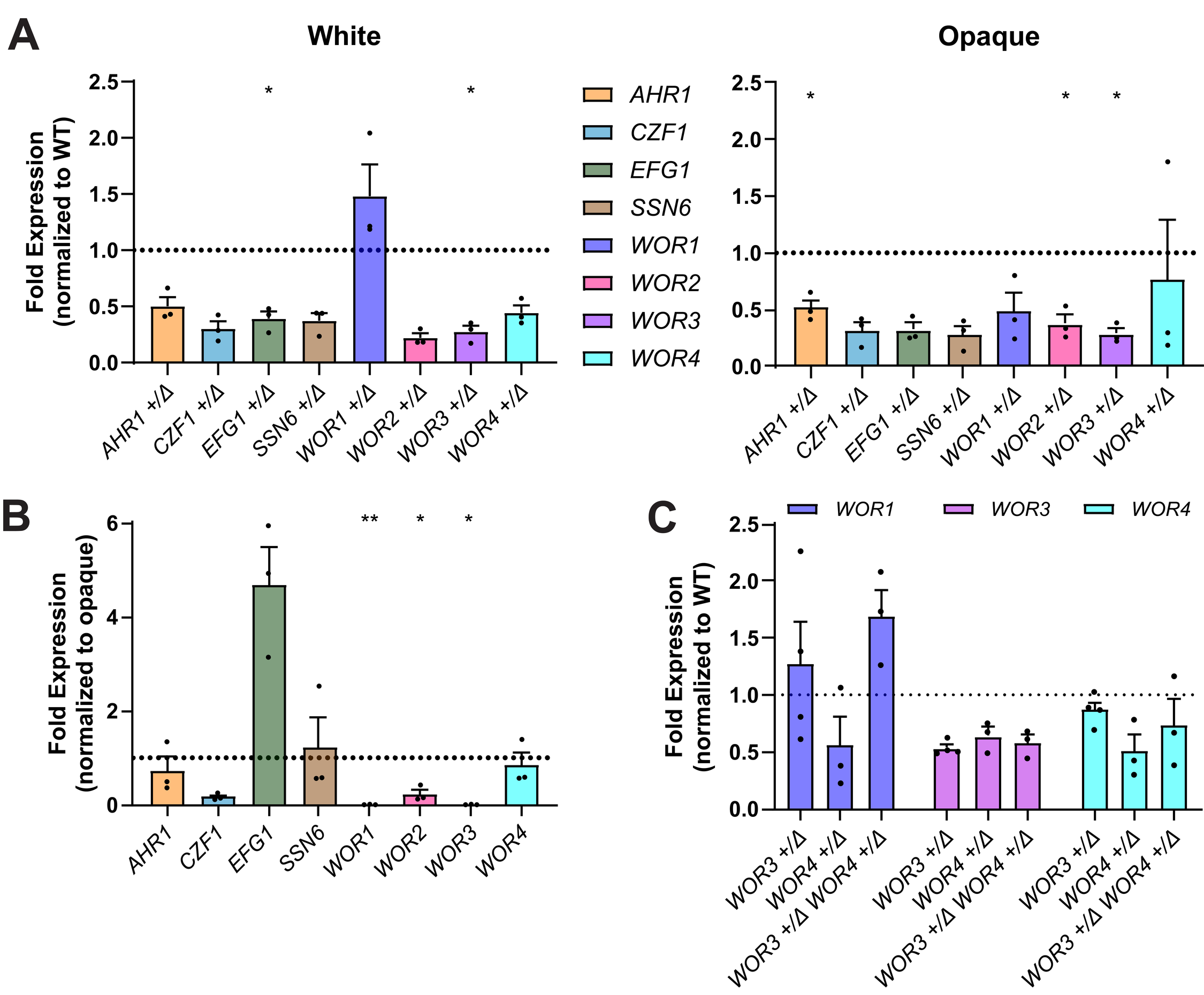

Supplement: S2 Fig — (A-C) WT and TF heterozygote strains were grown to mid-log phase in liquid SCD before RNA was harvested for qRT-PCR analysis. Mean normalized expression is shown. Black dots indicate biological replicates, error bars show SEM, and the dotted line corresponds to gene expression in the WT control. (A) TF gene expression from corresponding TF single heterozygotes is expressed relative to ACT1 and normalized to WT in both white (left) and opaque (right) cell types. Statistical analysis was performed using a two-tailed Student’s t-test. *P < 0.05. (B) TF gene expression from WT white cells is expressed relative to ACT1 and normalized to the WT opaque cells. Statistical analysis was performed using a two-tailed Student’s t-test, in which the fold expression was compared between the white and opaque cell states. *P < 0.05; **P < 0.01. (C) WOR1, WOR3, and WOR4 gene expression is shown (relative to ACT1 and normalized to WT opaque) in opaque WOR3 and WOR4 single heterozygotes and opaque WOR3 WOR4 double heterozygotes. (S2_Fig.TIF) [file pgen.1011810.s002.tif]

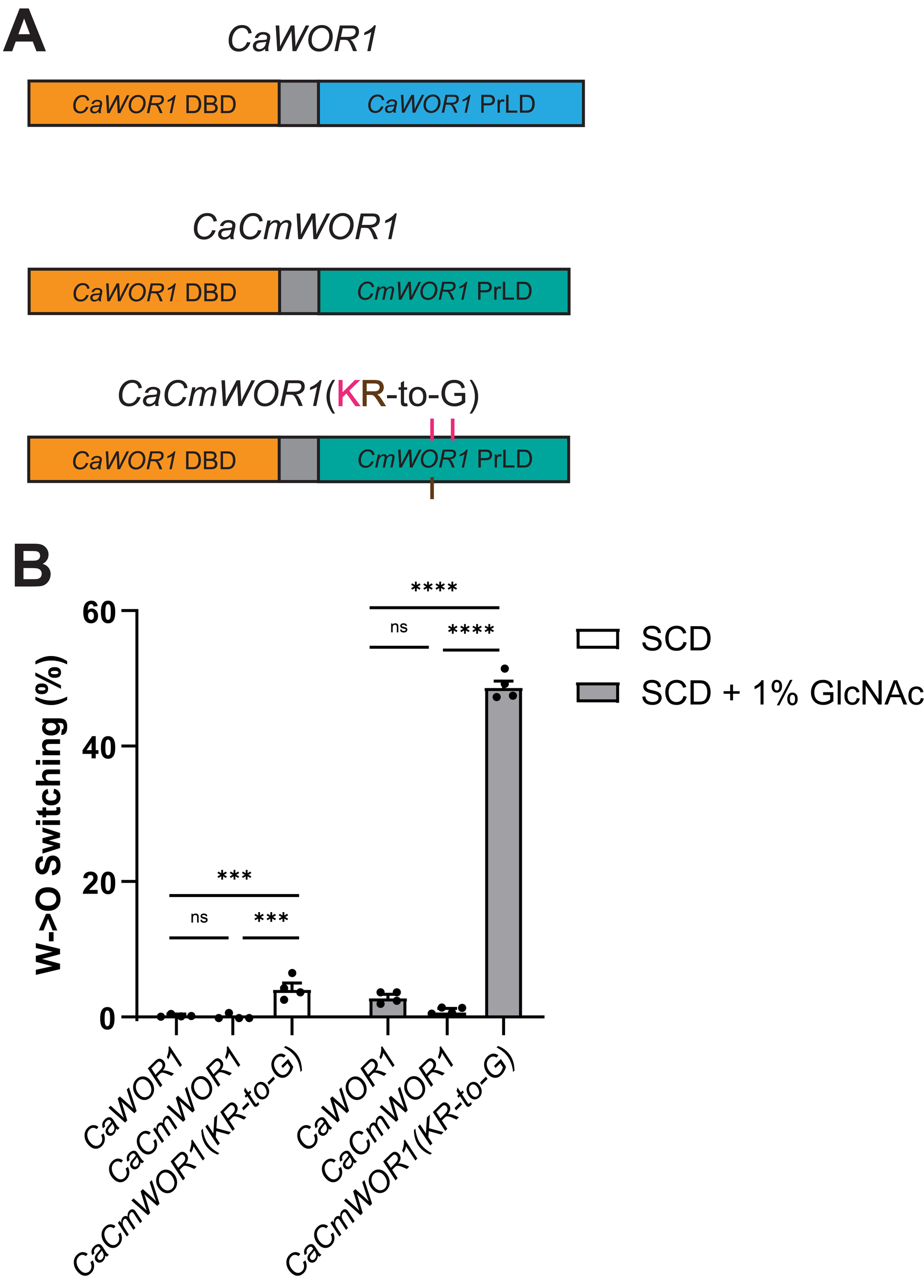

Supplement: S3 Fig — (A) Genotypic differences between CaWOR1, CaCmWOR1, and CaCmWOR1(KR-to-G). All three constructs contain the C. albicans WOR1 DNA binding domain (DBD; orange). CaWOR1 contains the C. albicans WOR1 PrLD (blue) while CaCmWOR1 constructs contain the C. maltosa WOR1 PrLD (light cyan). (B) All three constructs were grown on SCD (white) or SCD supplemented with 1% GlcNAc (gray) and switching frequencies determined after growth at 22°C for 7 days. Black dots indicate biological replicates and error bars show SEM. Statistical analysis was performed using ordinary one-way ANOVA with Dunnett’s multiple-comparison test, in which normalized switching frequencies were compared between each strain. ***P < 0.001; ****P < 0.0001; ns = not significant. (S3_Fig.TIF) [file pgen.1011810.s003.tif]

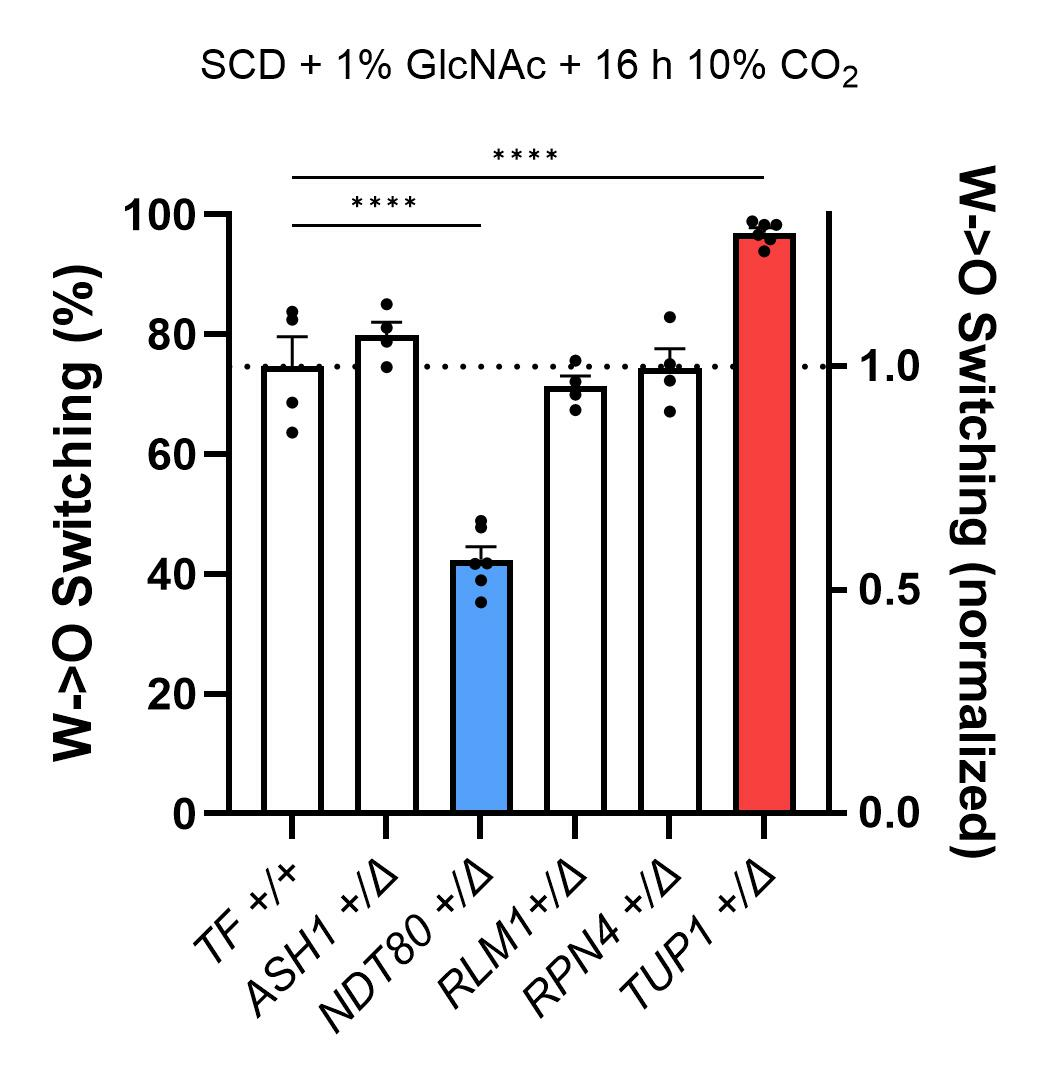

Supplement: S4 Fig — Strains were grown on SCD + 1% GlcNAc + 16 h 10% CO2 before outgrowth on normoxia. Switching frequencies were determined after growth at 25°C for 7 days. Mean white-to-opaque switching percentages are shown on the left y-axis and mean normalized white-to-opaque switching frequencies are shown on the right y-axis relative to the TF + / + control from same-day experiments. Black dots indicate biological replicates and error bars show SEM. Statistical analysis was performed using ordinary one-way ANOVA with Dunnett’s multiple-comparison test, in which heterozygote switching frequencies were compared to the WT strain. ****P < 0.0001. (S4_Fig.TIF) [file pgen.1011810.s004.tif]

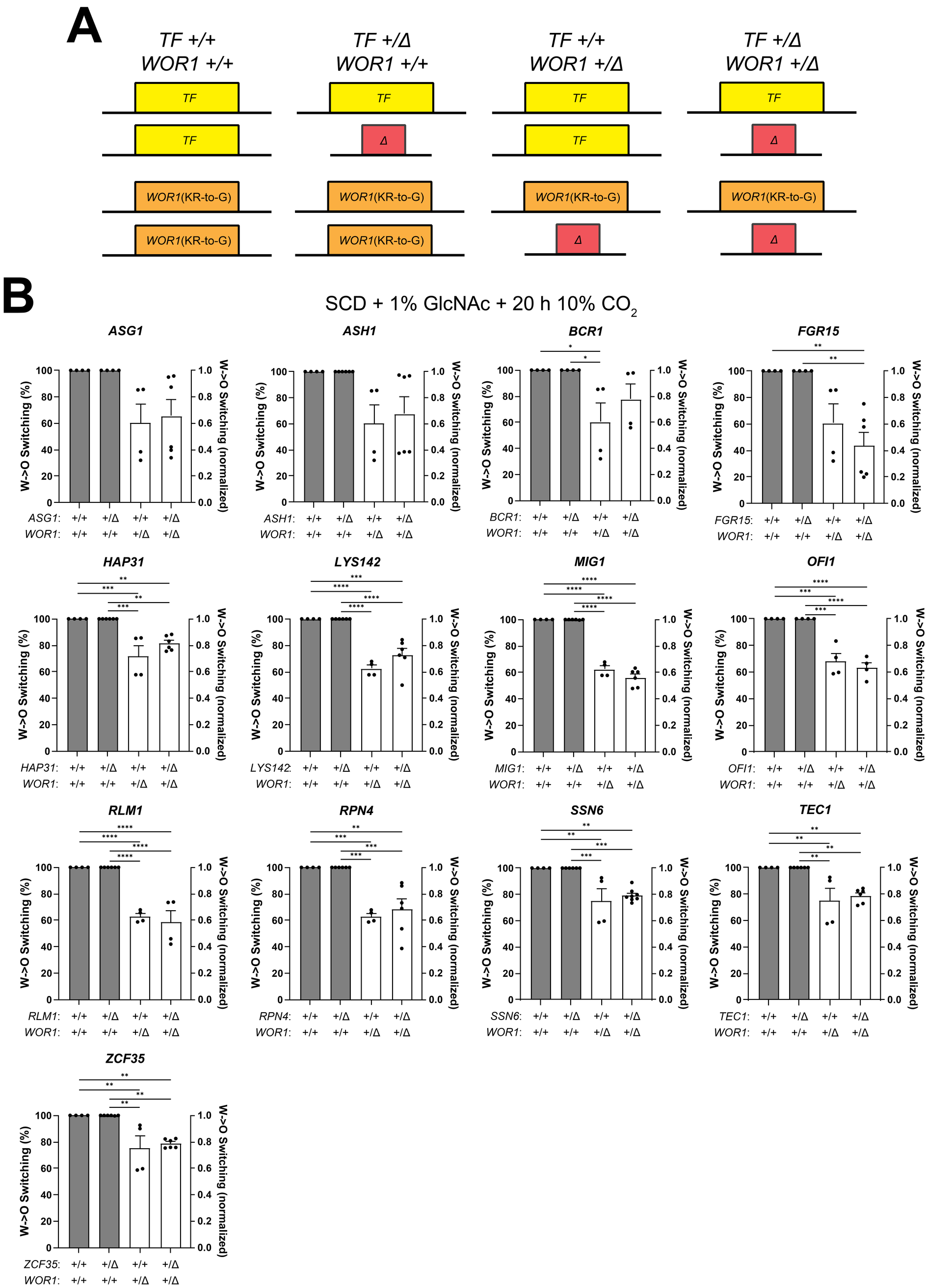

Supplement: S5 Fig — (A) Genotypes of the strains used. (B) WT, single heterozygotes and double heterozygotes in the WOR1(KR-to-G) strain background were grown on SCD + 1% GlcNAc in 10% CO2 for 20 h before outgrowth under normoxia. Switching frequencies were determined after growth at 25°C for 7 days. Mean white-to-opaque switching percentages are shown on the left y-axis and mean normalized white-to-opaque switching frequencies are shown on the right y-axis relative to WT from same-day experiments. Black dots indicate biological replicates and error bars show SEM. WOR1 single heterozygotes and double heterozygotes are shown in white; WT and TF single heterozygotes are shown in gray. Statistical analysis was performed using ordinary one-way ANOVA with Dunnett’s multiple-comparison test, in which switching frequencies were compared between each strain. *P < 0.05; **P < 0.01; ***P < 0.001; ****P < 0.0001. (S5_Fig.TIF) [file pgen.1011810.s005.tif]

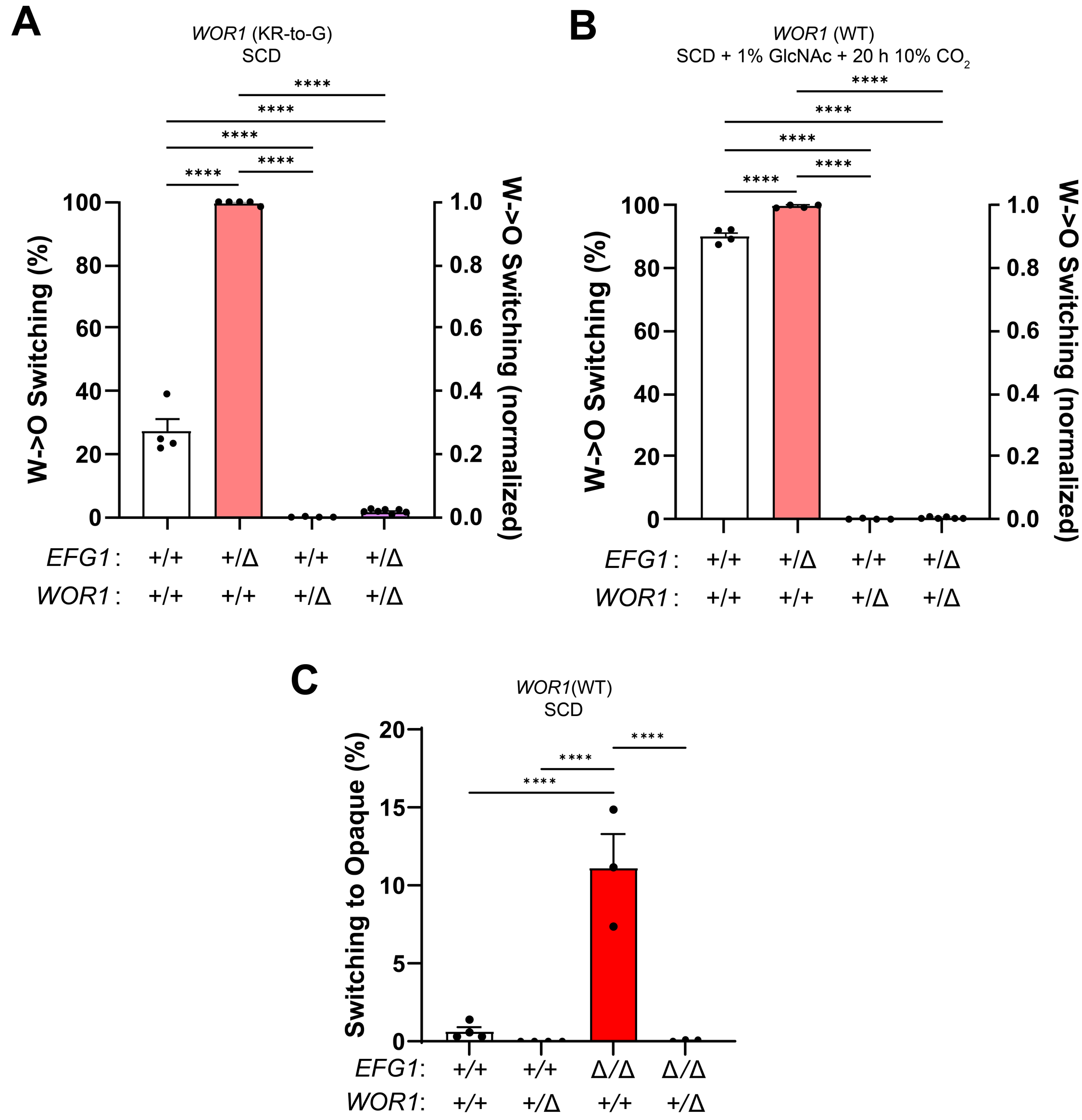

Supplement: S6 Fig — (A) WT, single, and double heterozygote strains in the WOR1(KR-to-G) background were grown on SCD. (B) WT, single, and double heterozygote strains in the WOR1(WT) background were grown on SCD + 1% GlcNAc + 20 h 10% CO2 before outgrowth in normoxia. (C) WT and WOR1 + /Δ (white cells) or efg1Δ/Δ and efg1Δ/ΔWOR1 + /Δ (gray cells) were grown on SCD. In A-C, switching frequencies were determined after growth at 25°C for 7 days. For A and B, mean white-to-opaque switching percentages are shown on the left y-axis and mean normalized white-to-opaque switching frequencies are shown on the right y-axis relative to the WT control from same-day experiments. For C, mean percentages of switching to the opaque state are shown on the left y-axis. Black dots indicate biological replicates and error bars show SEM. Statistical analysis was performed using ordinary one-way ANOVA with Dunnett’s multiple-comparison test, in which switching frequencies of each strain were compared to each other. ****P < 0.0001. (S6_Fig.TIF) [file pgen.1011810.s006.tif]

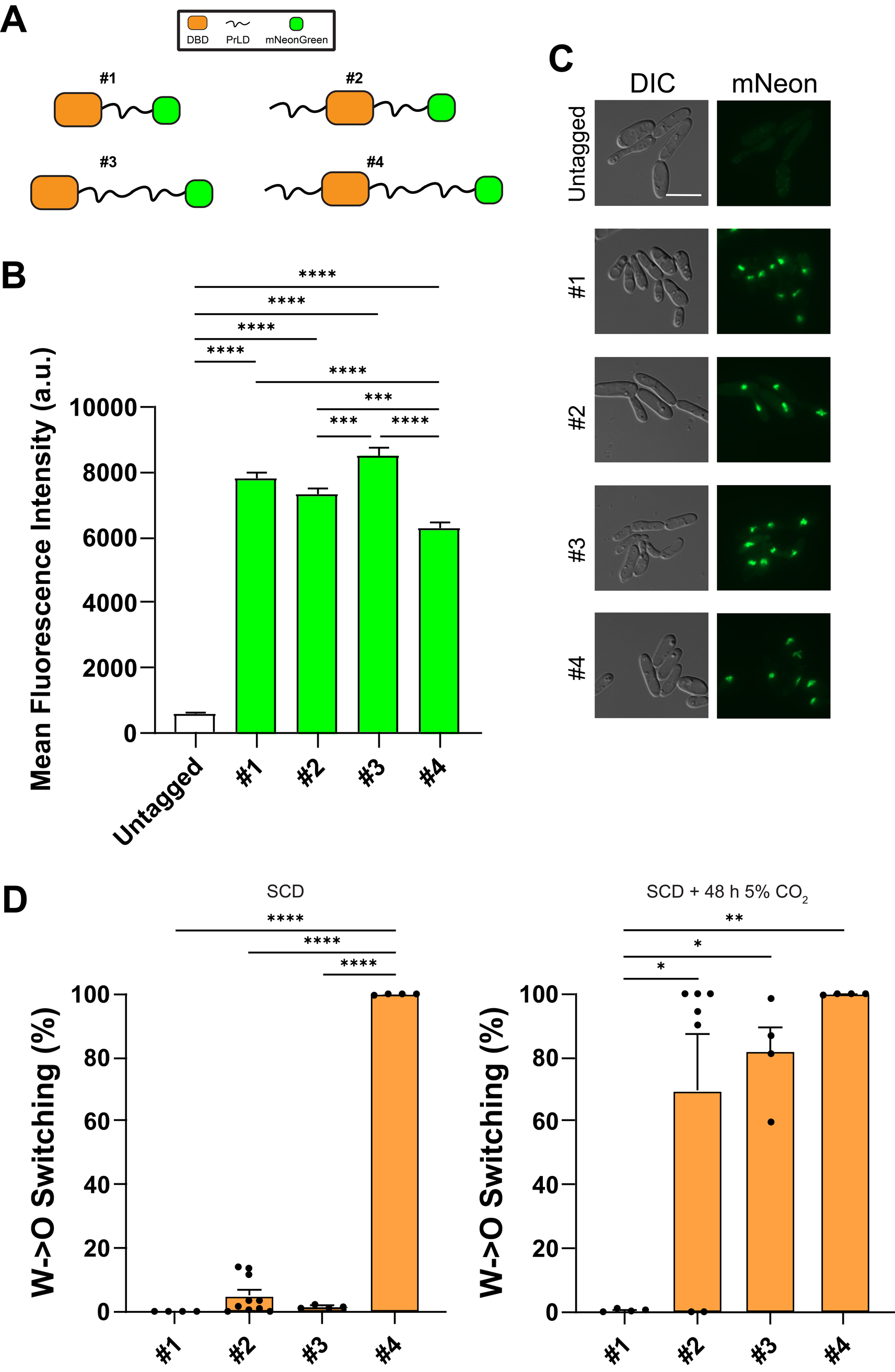

Supplement: S7 Fig — (A) Wor1 PLD variants were C-terminal tagged with mNeonGreen. (B) Wor1-mNeonGreen levels were quantified in opaque cells. Mean mNeonGreen expression levels are shown with error bars representing SEM. Statistical analysis was performed using ordinary one-way ANOVA with Dunnett’s multiple-comparison test, in which normalized switching frequencies were compared between each strain. *P < 0.05; **P < 0.01; ***P < 0.001; ****P < 0.0001. (C) Representative images of untagged or mNeonGreen-tagged opaque cells. Scale bar, 10 μm. (D) Strains were grown on SCD (left) or SCD + 48 h 5% CO2 before outgrowth in normoxia (right). Switching frequencies were determined after growth at 25°C for 7 days. Mean white-to-opaque switching percentages are shown. Black dots indicate biological replicates and error bars show SEM. Statistical analysis was performed using ordinary one-way ANOVA with Dunnett’s multiple-comparison test, in which switching frequencies of each strain were compared to each other. *P < 0.05; **P < 0.01; ****P < 0.0001. (S7_Fig.TIF) [file pgen.1011810.s007.tif]

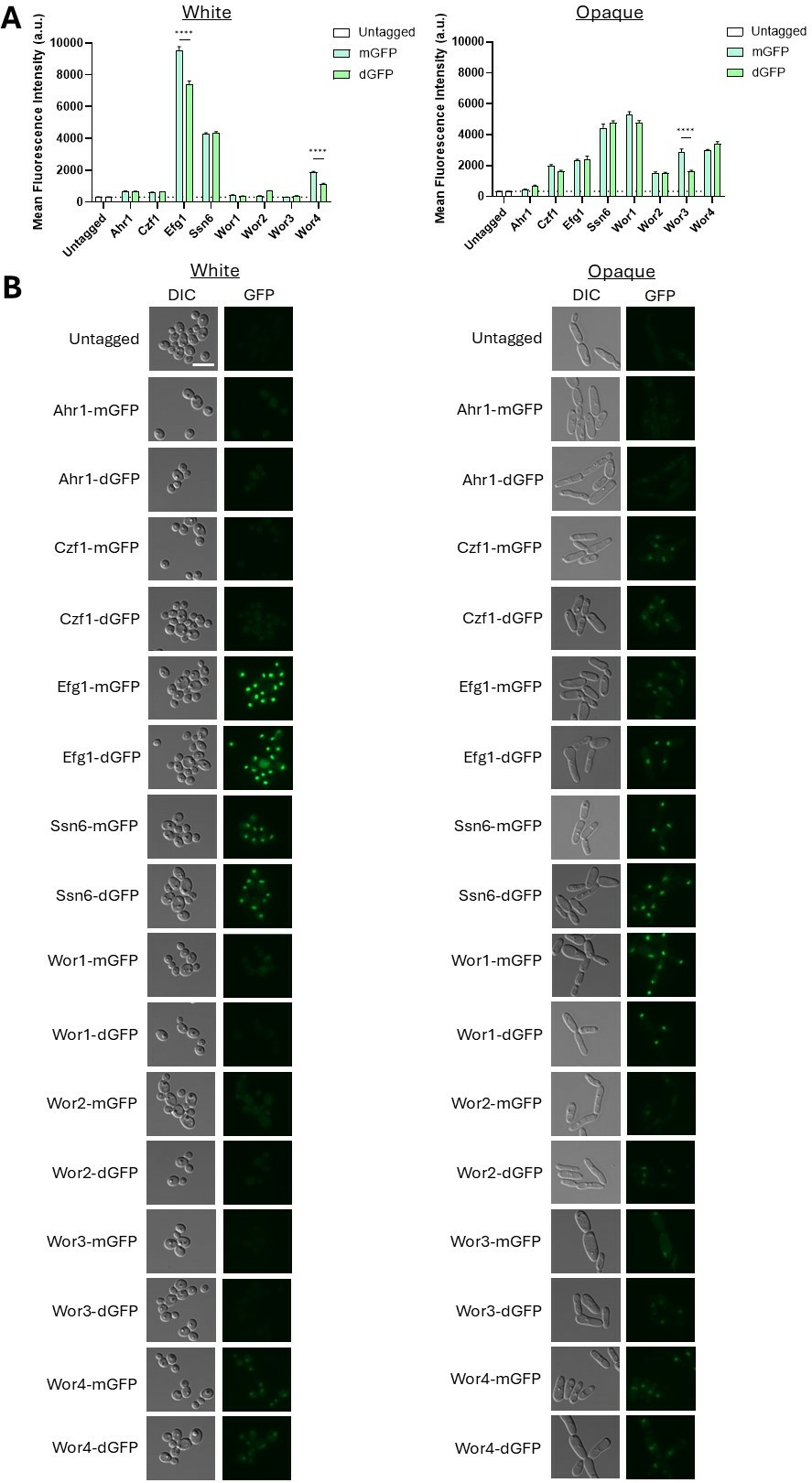

Supplement: S8 Fig — (A) TF-GFP levels were quantified in white and opaque cells. Cells were untagged (white bars) or tagged with mGFP (light cyan bars) or with dGFP (light green bars). Mean GFP expression levels are shown with error bars representing SEM, and the dotted line corresponding to the untagged control. Statistical analysis was performed using a two-tailed Student’s t-test in which mean GFP expression was compared between mGFP and dGFP for each TF. ****P < 0.0001. (B) Representative images of untagged cells or cells with both alleles of a TF tagged with mGFP or dGFP. Images are representative of two independent experimental replicates. Scale bar, 10 μm. (S8_Fig.TIF) [file pgen.1011810.s008.tif]

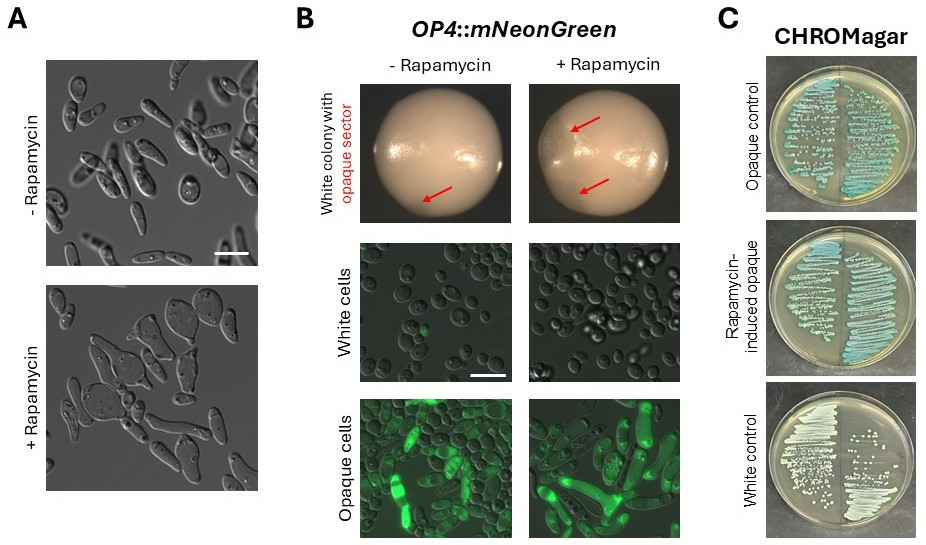

Supplement: S9 Fig — (A) Opaque cells from C. albicans WOR1-FRB/WOR1-RBP1 strain imaged in DIC. Scale bar, 10 μm. (B) White colonies with opaque sectors (denoted by red arrows) and corresponding white and opaque cells from a WOR1-FRB/WOR1-RBP1 strain with an OP4::mNeonGreen reporter. Scale bar, 10 μm. (C) WOR1-FRB/WOR1-RBP1 opaque and white cells cultured on CHROMagar at 22°C for six days. (S9_Fig.TIF) [file pgen.1011810.s009.tif]

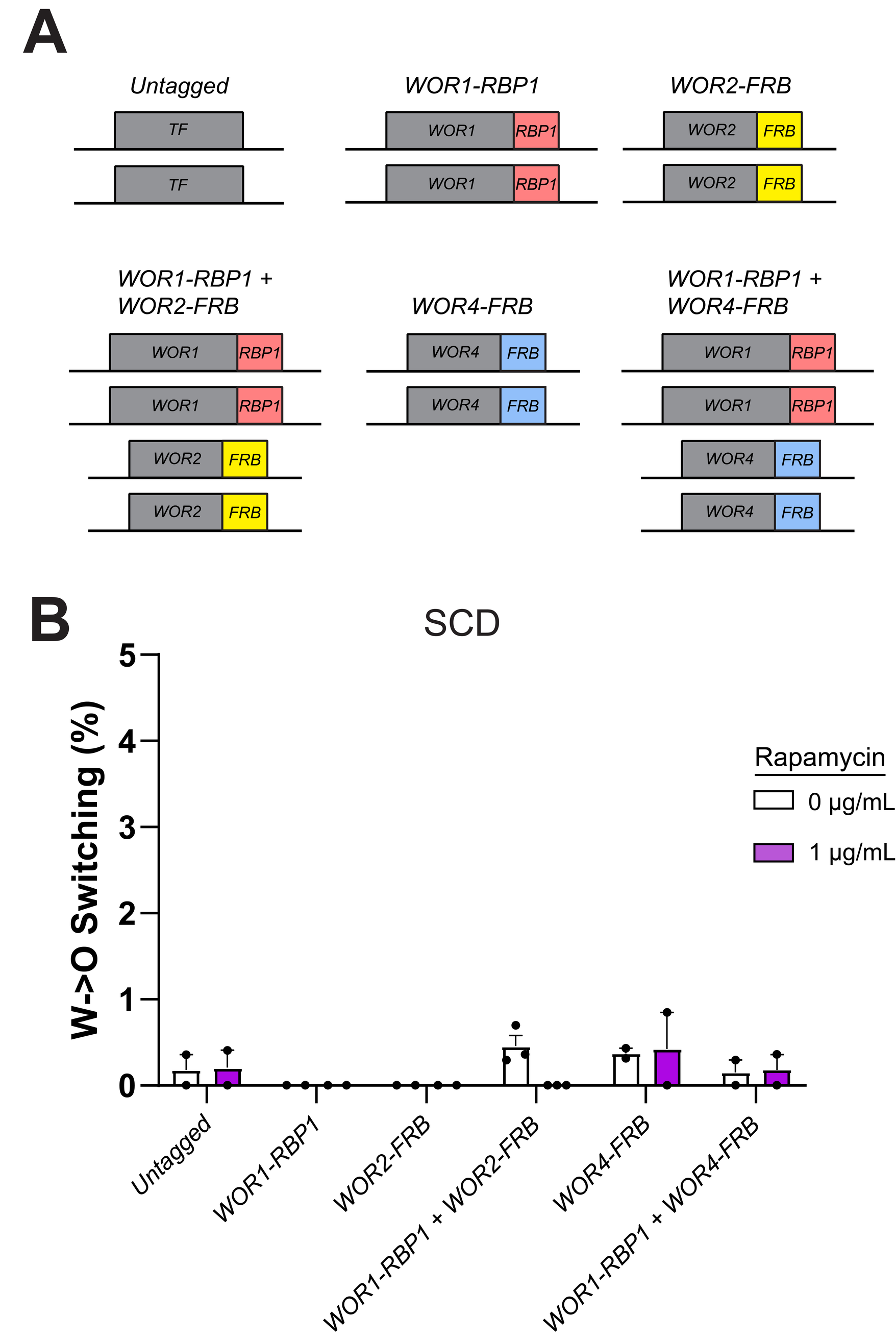

Supplement: S10 Fig — (A) Genotypes at WOR1, WOR2, and WOR4 loci in the six C. albicans strains tested. (B) Strains were grown on SCD medium at 22°C with 1 μg/mL rapamycin or with a vehicle control for 7 days. Mean white-to-opaque switching percentages are shown; black dots indicate biological replicates, and error bars show SEM. (S10_Fig.TIF) [file pgen.1011810.s010.tif]
